# Supplementary material for: A methodological framework for exploring SME finance with SAFE data
Source: PLoS One. 2024 Aug 29;19(8):e0307361. doi: 10.1371/journal.pone.0307361 (PMC11361696; doi:10.1371/journal.pone.0307361)
Supplement: S1 Table — (DOCX) [file pone.0307361.s002.docx]

**S1 Table. Variables, definition and data source**

| Variable | Data Source | Definition |
| --- | --- | --- |
| **Dependent variable** |  |  |
| $\boldsymbol{P}\left( \boldsymbol{CreditConstrained}_{\boldsymbol{i,c,t}}\boldsymbol{=1} \vert\boldsymbol{x} \right)$ | ECB/EC Survey on the access to finance of enterprises (SAFE)  Q7a, Q7b | Binary variable = 1 if the firm reported (i) to have applied for bank loans in the previous six months but was rejected (Credit Denied) or (ii) to have applied but received less than 75% of its demand (Rationed) or (iii) to have refused credit because it was offered at a too high cost (Refused due to high cost) or (iv) not to have applied because of possible rejection (Discouraged). 0 = if the firm reported (i) to have applied for bank loans in the previous six months and they received everything or (ii) they received 75 percent or more of their demand. |
| **Independent variable H1** |  |  |
| $\mathbf{H}\boldsymbol{1}\boldsymbol{\gamma}^{\boldsymbol{'}}\boldsymbol{DebttoAssets}_{\boldsymbol{i,c,t}}\boldsymbol{*}\boldsymbol{MP}_{\boldsymbol{c,t-2}}$ |  |  |
| Debt to Assets increased | ECB/EC SAFE Q2 | = 1 if the firm’s debt to assets increased in the past 6 months, 0 if it remained unchanged or decreased |
| MPc,t-2 = ECB BS Assets (million) | ECB Statistical Data Warehouse | Continuous variable, monthly, total ECB assets (after subtracting the autonomous factors that are beyond the direct control of the ECB including banknotes in circulation and government balances) from individual central bank balance sheet for stressed countries, following Peydro et al. (81). Monthly data averaged over half years ending in March and September. |
|  |  |  |
| **Independent variable H2** |  |  |
| **H2** $\boldsymbol{MP}_{\boldsymbol{c,t-2}}\boldsymbol{+}\boldsymbol{\gamma}^{\boldsymbol{'}}\boldsymbol{FirmRisk}_{\boldsymbol{i,c,t}}$ |  |  |
| Where ${FirmRisk}_{i,c,t}$*=* |  |  |
| Profit decreased | ECB/EC SAFE Q2 | = 1 if the firm's profit decreased in the past 6 months, 0 if it remained unchanged or increased |
| Credit history deteriorated | ECB/EC SAFE Q11 | = 1 if the firm's credit history deteriorated in the previous 6 months, 0 if it remained the unchanged or improved |
| Own capital deteriorated | ECB/EC SAFE Q11 | = 1 if the firm's own capital deteriorated in the previous 6 months, 0 if it remained unchanged or improved |
| Own outlook deteriorated | ECB/EC SAFE Q11 | = 1 if the firm's own outlook deteriorated in the previous 6 months, 0 if it remained unchanged or improved |
| Innovation | ECB/EC SAFE Q1 | = 1 if the innovated (in terms of new or improved product, new or improved production process, new organisation of management, new way of selling goods or services) in the previous 6 months, 0 if did not |
| $\boldsymbol{X}_{\boldsymbol{i,c,t}}$ **= Firm controls** |  |  |
| Micro | ECB/EC SAFE  QD1 | = 1 if the firm has between 1 and 9 employees, 0 otherwise |
| Micro | ECB/EC SAFE  QD1 | = 1 if the firm has between 1 and 9 employees, 0 otherwise |
| Small | ECB/EC SAFE  QD1 | = 1 if the firm has between 10 and 49 employees, 0 otherwise |
| Medium | ECB/EC SAFE  QD1 | = 1 if the firm has between 50 and 249 employees, 0 otherwise |
| More than 10 years | ECB/EC SAFE  QD5 | = 1 if the firm is 10+ years old, 0 otherwise |
| Between 5 and 10 yrs | ECB/EC SAFE  QD5 | = 1 if the firm is between 5 and 10 years old, 0 otherwise |
| Between 2 and 5 yrs | ECB/EC SAFE  QD5 | = 1 if the firm is between 2 and 5 years old, 0 otherwise |
| Less than 2 yrs | ECB/EC SAFE  QD5 | = 1 if the firm is less than 2 years old, 0 otherwise |
| Stand-alone firm | ECB/EC SAFE  QD2 | = 1 if the firm is an autonomous profit-oriented enterprise, 0 otherwise |
| Individual or family owned | ECB/EC SAFE  QD2 | = 1 if the firm's owner is an individual or a family, 0 otherwise |
| Turnover up to 2mn | ECB/EC SAFE  QD4 | = 1 if the firm's annual turnover is less than €2 mln, 0 otherwise |
| Turnover between 2 and 10mn | ECB/EC SAFE  QD4 | = 1 if the firm's annual turnover is between €2 mln. and €10 mln, 0 otherwise |
| Turnover between 10 and 50mn | ECB/EC SAFE  QD4 | = 1 if the firm's annual turnover is between €10 mn. and €50 mn, 0 otherwise |
| Industry | ECB/EC SAFE  QD3 | = 1 if the firm's main activity is in industry, 0 otherwise |
| Construction | ECB/EC SAFE  QD3 | = 1 if the firm's main activity is in construction, 0 otherwise |
| Wholesale or Retail Trade | ECB/EC SAFE  QD3 | = 1 if the firm's main activity is in wholesale or retail trade, 0 otherwise |
| Services | ECB/EC SAFE  QD3 | = 1 if the firm's main activity is services, 0 otherwise |
| $\boldsymbol{Macro}_{\boldsymbol{c,t-2}}$***= Macroeconomic controls*** |  |  |
| Unemployment | Eurostat | Continuous variable, unemployment rate (share of active population), seasonally adjusted. The unemployment rate is the number of people unemployed expressed as a share of the labour force. The labour force is the total number of people employed and unemployed. Quarterly data averaged over half years ending in March and September and expressed as decimals. |
| Inflation | Eurostat | Continuous variable, inflation rate measured by HICP monthly data (annual rate of change). Average of monthly inflation rate data over half years ending in March and September and expressed as decimals. |
| $\boldsymbol{BankCh}_{\boldsymbol{c,t-2}}\boldsymbol{=}$***Bank controls*** |  |  |
| Non-performing loans | IMF Financial Soundness Indicators | Continuous variable, quarterly non-performing loans as a share of total gross loans averaged over half years ending in March and September and expressed as decimals. |
| Regulatory capital to risk-weighted assets ratio | IMF Financial Soundness Indicators | Continuous variable, quarterly regulatory tier 1 capital as a share of risk weighted assets averaged over half years ending in March and September and expressed as decimals. |

Source: Finnegan and Kapoor (2023). Mn = million.
