# Supplementary material for: A methodological framework for exploring SME finance with SAFE data
Source: PLoS One. 2024 Aug 29;19(8):e0307361. doi: 10.1371/journal.pone.0307361 (PMC11361696; doi:10.1371/journal.pone.0307361)
Supplement: S3 Table — (DOCX) [file pone.0307361.s004.docx]

**S3 Table. H1, MPt-2 X Debt to Assets increased, Probability of being credit constrained on monetary policy and debt to assets, probit versus logit.**

| Credit constrained | (1) | | (2) | (3) | (4) | (5) | | | (6) | | (7) | | (8) | |
| --- | --- | --- | --- | --- | --- | --- | --- | --- | --- | --- | --- | --- | --- | --- |
| Variables |  | |  |  |  |  | | |  | |  | |  | |
|  |  | | Probit |  |  |  | | |  | | Logit | |  | |
|  |  | |  |  |  |  | | |  | |  | |  | |
| MP_t−2_ | -0.00886 | | -0.0181 | -0.0327 | -0.0309 | -0.0107 | | | -0.0207 | | -0.0342 | | -0.0316 | |
|  | (0.0240) | | (0.0263) | (0.0299) | (0.0286) | (0.0238) | | | (0.0261) | | (0.0296) | | (0.0284) | |
| Debt to assets increased | -0.546*** | | -0.551*** | -0.551*** | -0.460*** | -0.541*** | | | -0.546*** | | -0.545*** | | -0.468*** | |
|  | (0.136) | | (0.136) | (0.136) | (0.130) | (0.135) | | | (0.134) | | (0.134) | | (0.129) | |
| MP_t-2_ x Debt to assets increased | 0.0491*** | | 0.0495*** | 0.0495*** | 0.0417*** | 0.0487*** | | | 0.0491*** | | 0.0490*** | | 0.0423*** | |
|  |  | |  |  |  |  | | |  | |  | |  | |
|  | (0.0114) | | (0.0114) | (0.0114) | (0.0109) | (0.0113) | | | (0.0113) | | (0.0112) | | (0.0108) | |
| **Bank characteristic variables** |  |  |  |  |  |  |  | |  | |  | |  | |
| Non-performing loans_t-2_ |  | | -0.00234 | -0.00281 | -0.00256 |  | | | -0.00248 | | -0.00293 | | -0.00251 | |
|  |  | | (0.00188) | (0.00211) | (0.00200) |  | | | (0.00189) | | (0.00211) | | (0.00200) | |
| Tier 1 Capital Ratio_t-2_ |  | | -0.00238 | -0.00506 | -0.00418 |  | | | -0.00284 | | -0.00528 | | -0.00407 | |
|  |  | | (0.00599) | (0.00652) | (0.00617) |  | | | (0.00608) | | (0.00657) | | (0.00618) | |
| **Macroeconomic variables** |  |  |  |  |  |  | |  | |  | |  | |  |
| Unemployment_t-2_ |  | |  | 0.00148 | 0.00140 |  | | |  | | 0.00143 | | 0.00112 | |
|  |  | |  | (0.00170) | (0.00164) |  | | |  | | (0.00170) | | (0.00165) | |
| Inflation_t-2_ |  | |  | 0.0135 | 0.0113 |  | | |  | | 0.0127 | | 0.0101 | |
|  |  | |  | (0.0143) | (0.0138) |  | | |  | | (0.0142) | | (0.0137) | |
| **Firm characteristic variables** |  |  |  |  |  |  |  | |  | |  | |  | |
| Micro |  | |  |  | 0.0932*** |  | | |  | |  | | 0.0928*** | |
|  |  | |  |  | (0.0180) |  | | |  | |  | | (0.0180) | |
| Small |  | |  |  | 0.0114 |  | | |  | |  | | 0.0130 | |
|  |  | |  |  | (0.0153) |  | | |  | |  | | (0.0157) | |
| Trade |  | |  |  | -0.0366 |  | | |  | |  | | -0.0396 | |
|  |  | |  |  | (0.0289) |  | | |  | |  | | (0.0321) | |
| Industry |  | |  |  | -0.0832*** |  | | |  | |  | | -0.0849*** | |
|  |  | |  |  | (0.0285) |  | | |  | |  | | (0.0281) | |
| Less than 2yrs |  | |  |  | 0.0867* |  | | |  | |  | | 0.0892* | |
|  |  | |  |  | (0.0519) |  | | |  | |  | | (0.0497) | |
| Between 2 and 5yrs |  | |  |  | 0.126*** |  | | |  | |  | | 0.123*** | |
|  |  | |  |  | (0.0257) |  | | |  | |  | | (0.0252) | |
| Between 5 and 10yrs |  | |  |  | 0.0162 |  | | |  | |  | | 0.0172 | |
|  |  | |  |  | (0.0159) |  | | |  | |  | | (0.0158) | |
| Turnover up to 2mn |  | |  |  | 0.303*** |  | | |  | |  | | 0.323*** | |
|  |  | |  |  | (0.0377) |  | | |  | |  | | (0.0432) | |
| Turnover 2-10mn |  | |  |  | 0.180*** |  | | |  | |  | | 0.202*** | |
|  |  | |  |  | (0.0364) |  | | |  | |  | | (0.0422) | |
| Turnover 10-50mn |  | |  |  | 0.0685* |  | | |  | |  | | 0.0819* | |
|  |  | |  |  | (0.0365) |  | | |  | |  | | (0.0427) | |
| Individual or family owned |  | |  |  | -0.00874 |  | | |  | |  | | -0.00628 | |
|  |  | |  |  | (0.0153) |  | | |  | |  | | (0.0156) | |
| Stand-alone firm |  | |  |  | -0.0966*** |  | | |  | |  | | -0.0990*** | |
|  |  | |  |  | (0.0215) |  | | |  | |  | | (0.0222) | |
| Observations | 8,777 | | 8,777 | 8,777 | 8,668 | 8,777 | | | 8,777 | | 8,777 | | 8,668 | |
| Country*Sector FE | Yes | | Yes | Yes | Yes | Yes | | | Yes | | Yes | | Yes | |
| Time FE | Yes | | Yes | Yes | Yes | Yes | | | Yes | | Yes | | Yes | |
| Bank Controls | No | | Yes | Yes | Yes | No | | | Yes | | Yes | | Yes | |
| Macro Controls | No | | No | Yes | Yes | No | | | No | | Yes | | Yes | |
| Other Firm Controls | No | | No | No | Yes | No | | | No | | No | | Yes | |
| Goodness of Fit |  | |  |  |  |  | | |  | |  | |  | |
| Mc Fadden’s Pseudo R2 | 0.1133 | | 0.1134 | 0.114 | 0.181 | 0.113 | | | 0.113 | | 0.114 | | 0.181 | |
| Mc Fadden’s Adjusted Pseudo R2 | 0.107 | | 0.107 | 0.107 | 0.171 | 0.107 | | | 0.107 | | 0.107 | | 0.171 | |
| Percentage Correctly Predicted (PCP) | 0.709 | | 0.709 | 0.708 | 0.732 | 0.709 | | | 0.709 | | 0.709 | | 0.732 | |
| Percentage Reduction in Error (PRE) | 0.221 | | 0.220 | 0.219 | 0.282 | 0.221 | | | 0.221 | | 0.221 | | 0.283 | |
| Expected PCP Herron | 0.601 | | 0.602 | 0.601 | 0.637 | 0.601 | | | 0.602 | | 0.602 | | 0.637 | |
| Expected PRE Herron | 0.148 | | 0.148 | 0.147 | 0.223 | 0.148 | | | 0.149 | | 0.149 | | 0.225 | |
| BIC | -69084.103 | | -69067.545 | -69050.67 | -68706.826 | -69084.417 | | | -69068.063 | | -69051.092 | | -68713.767 | |
| AIC | 1.180 | | 1.180 | 1.180 | 1.096 | 1.180 | | | 1.180 | | 1.180 | | 1.095 | |
| Area under ROC curve | 0.7041 | | 0.7043 | 0.7043 | 0.7672 | 0.7041 | | | 0.7043 | | 0.7044 | | 0.7678 | |
| Residual deviance | 10283.216 | |  | 10280.329 | 9390.633 | 10282.901 | | | 10281.095 | | 10279.907 | | 9383.692 | |
| Statistical inference |  | |  |  |  |  | | |  | |  | |  | |
| Wald Chi2 X2 | 1177.66 | | 1169.02 | 1170.55 | 1723.53 | 1065.15 | | | 1054.08 | | 1055.78 | | 1495.30 | |
| Prob > Chi2 | 0.0000 | | 0.0000 | 0.0000 | 0.0000 | 0.0000 | | | 0.0000 | | 0.0000 | | 0.0000 | |
| LR | 1314.043 | | 1315.645 | 1316.929 | 2016.236 | 1314.357 | | | 1316.163 | | 1317.352 | | 2068.177 | |
| Prob > LR | 0.000 | | 0.000 | 0.000 | 0.0000 | 0.0000 | | | 0.0000 | | 0.0000 | | 0.0000 | |

The probability of being credit constrained is the dependent variable for stressed countries with probit in columns (1)-(4) and for stressed countries with logit in columns (5)-(8). Reported estimates are conditional marginal effects drawn from both probit and logit regression models with sample selection for the pooled sample of SMEs. MP_t−2_ is one-year lag (equivalent to two survey waves). Debt-to-assets increased is a categorical variable which equals 1 if the firm’s debt-to-assets increased, and 0 if it remained the same or decreased in the previous six months. Bank controls (non-performing loans and tier-1 capital ratio) and macro controls (inflation and unemployment) are lagged by one-year (equivalent to two survey waves). Robust standard errors are in the parentheses. ***, **, * represent significance at 1%, 5% and 10%, respectively.
