# Supplementary material for: A methodological framework for exploring SME finance with SAFE data
Source: PLoS One. 2024 Aug 29;19(8):e0307361. doi: 10.1371/journal.pone.0307361 (PMC11361696; doi:10.1371/journal.pone.0307361)
Supplement: S4 Table — (DOCX) [file pone.0307361.s005.docx]

**S4 Table. H2, LPM, Probability that the implementation of UMP leads to risky firms being less credit constrained**

| VARIABLES | (1) | (2) | (3) | (4) | (5) | (6) | (7) | (8) | (9) | (10) |
| --- | --- | --- | --- | --- | --- | --- | --- | --- | --- | --- |
| MP_t−2_ | -0.00525 | -0.00537 | -0.0147 | -0.0147 | -0.00410 | -0.00626 | -0.0277 | -0.0258 | -0.0178 | -0.0176 |
|  | (0.0316) | (0.0304) | (0.0308) | (0.0297) | (0.0308) | (0.0298) | (0.0309) | (0.0298) | (0.0316) | (0.0302) |
| Profit decreased | 0.119 | 0.150 |  |  |  |  |  |  |  |  |
|  | (0.141) | (0.137) |  |  |  |  |  |  |  |  |
| MP_t−2_ X Profit decreased | 0.00498 | -0.00112 |  |  |  |  |  |  |  |  |
|  | (0.0118) | (0.0114) |  |  |  |  |  |  |  |  |
| Credit history deteriorated |  |  | -0.130 | -0.108 |  |  |  |  |  |  |
|  |  |  | (0.209) | (0.207) |  |  |  |  |  |  |
| MP_t-2_ x Credit history |  |  | 0.0368** | 0.0308* |  |  |  |  |  |  |
|  |  |  | (0.0177) | (0.0175) |  |  |  |  |  |  |
| Own outlook deteriorated |  |  |  |  | 0.259* | 0.196 |  |  |  |  |
|  |  |  |  |  | (0.156) | (0.152) |  |  |  |  |
| MP_t-2_ x Own outlook |  |  |  |  | 0.00167 | 0.00378 |  |  |  |  |
|  |  |  |  |  | (0.0131) | (0.0127) |  |  |  |  |
| Own capital deteriorated |  |  |  |  |  |  | -0.188 | -0.184 |  |  |
|  |  |  |  |  |  |  | (0.190) | (0.188) |  |  |
| MP_t-2_ x Own capital |  |  |  |  |  |  | 0.0410** | 0.0353** |  |  |
|  |  |  |  |  |  |  | (0.0163) | (0.0162) |  |  |
| Innovation |  |  |  |  |  |  |  |  | -0.125 | -0.101 |
|  |  |  |  |  |  |  |  |  | (0.140) | (0.135) |
| MP_t-2_ x innovation |  |  |  |  |  |  |  |  | 0.0116 | 0.00984 |
|  |  |  |  |  |  |  |  |  | (0.0117) | (0.0113) |
| **Bank characteristic variables** |  |  |  |  |  |  |  |  |  |  |
| Non-performing loans_t-2_ | -0.0002 | 4.11e-05 | -0.000932 | -0.000718 | 0.00599*** | 0.00516** | -0.00109 | -0.00111 | -0.00226 | -0.00185 |
|  | (0.0020) | (0.00200) | (0.00209) | (0.00200) | (0.00208) | (0.00200) | (0.00208) | (0.00200) | (0.00212) | (0.00201) |
| Reg tier capital ratio_t-2_ | -0.00235 | -0.00238 | -0.00280 | -0.00283 | 0.00319 | 0.00199 | -0.00341 | -0.00338 | -0.00213 | -0.00236 |
|  | (0.0063) | (0.00606) | (0.00636) | (0.00607) | (0.00633) | (0.00606) | (0.00630) | (0.00604) | (0.00643) | (0.00609) |
| **Macroeconomics characteristic variables** |  |  |  |  |  |  |  |  | -0.125 | -0.101 |
| Unemployment_t-2_ | 0.00175 | 0.00165 | 0.00219 | 0.00208 | 0.00187 | 0.00176 | 0.00195 | 0.00172 | 0.00157 | 0.00145 |
|  | (0.0017) | (0.00166) | (0.00167) | (0.00163) | (0.00166) | (0.00163) | (0.00169) | (0.00165) | (0.00172) | (0.00167) |
| Inflation_t-2_ | 0.009 | 0.00895 | 0.0149 | 0.0123 | -0.00131 | -0.000372 | 0.0186 | 0.0159 | 0.0158 | 0.0130 |
|  | (0.0147) | (0.0143) | (0.0146) | (0.0142) | (0.0144) | (0.0140) | (0.0147) | (0.0142) | (0.0149) | (0.0144) |
| **Firm characteristic variables** |  |  |  |  |  |  |  |  | -0.125 | -0.101 |
| Micro |  | 0.0942*** |  | 0.0877*** |  | 0.0833*** |  | 0.0893*** |  | 0.102*** |
|  |  | (0.0180) |  | (0.0179) |  | (0.0177) |  | (0.0179) |  | (0.0181) |
| Small |  | 0.00947 |  | 0.00335 |  | 0.00148 |  | 0.00847 |  | 0.00895 |
|  |  | (0.0140) |  | (0.0138) |  | (0.0138) |  | (0.0139) |  | (0.0140) |
| Trade |  | -0.0444 |  | -0.0360 |  | -0.0455 |  | -0.0330 |  | -0.0378 |
|  |  | (0.0313) |  | (0.0313) |  | (0.0316) |  | (0.0315) |  | (0.0316) |
| Industry |  | -0.0919*** |  | -0.0742** |  | -0.0946*** |  | -0.0805*** |  | -0.0853*** |
|  |  | (0.0299) |  | (0.0299) |  | (0.0302) |  | (0.0300) |  | (0.0302) |
| Less than 2yrs |  | 0.125** |  | 0.0929 |  | 0.108* |  | 0.111* |  | 0.102* |
|  |  | (0.0602) |  | (0.0596) |  | (0.0612) |  | (0.0598) |  | (0.0622) |
| Between 2-5yrs |  | 0.137*** |  | 0.142*** |  | 0.125*** |  | 0.142*** |  | 0.137*** |
|  |  | (0.0277) |  | (0.0284) |  | (0.0276) |  | (0.0283) |  | (0.0282) |
| Between 5-10yrs |  | 0.0275 |  | 0.0242 |  | 0.0180 |  | 0.0233 |  | 0.0172 |
|  |  | (0.0172) |  | (0.0172) |  | (0.0170) |  | (0.0174) |  | (0.0173) |
| Turnover up to 2mn |  | 0.245*** |  | 0.244*** |  | 0.229*** |  | 0.247*** |  | 0.267*** |
|  |  | (0.0267) |  | (0.0262) |  | (0.0269) |  | (0.0262) |  | (0.0269) |
| Turnover between 2-10mn |  | 0.118*** |  | 0.120*** |  | 0.111*** |  | 0.128*** |  | 0.132*** |
|  |  | (0.0239) |  | (0.0233) |  | (0.0241) |  | (0.0232) |  | (0.0240) |
| Turnoverbetween10-50mn |  | 0.0372* |  | 0.0332 |  | 0.0244 |  | 0.0389* |  | 0.0382* |
|  |  | (0.0225) |  | (0.0219) |  | (0.0226) |  | (0.0218) |  | (0.0226) |
| Individual or family-owned |  | -0.0105 |  | -0.00992 |  | -0.00275 |  | -0.00840 |  | -0.00909 |
|  |  | (0.0143) |  | (0.0139) |  | (0.0140) |  | (0.0141) |  | (0.0143) |
| Stand-alone firm |  | -0.0842*** |  | -0.0819*** |  | -0.0863*** |  | -0.0794*** |  | -0.0864*** |
|  | (0.0210) | (0.0210) |  | (0.0202) |  | (0.0209) |  | (0.0206) |  | (0.0209) |
| Observations | 8,726 | 8,726 | 8,896 | 8,779 | 8,826 | 8,707 | 8,849 | 8,734 | 8,943 | 8,820 |
| Country*Sector FE | Yes | Yes | Yes | Yes | Yes | Yes | Yes | Yes | Yes | Yes |
| Time FE | Yes | Yes | Yes | Yes | Yes | Yes | Yes | Yes | Yes | Yes |
| Bank Controls | Yes | Yes | Yes | Yes | Yes | Yes | Yes | Yes | Yes | Yes |
| Macro Controls | Yes | Yes | Yes | Yes | Yes | Yes | Yes | Yes | Yes | Yes |
| Other Firm Controls | Yes | Yes | No | Yes | No | Yes | No | Yes | No | Yes |
| Goodness of fit (LPM) |  |  |  |  |  |  |  |  |  |  |
| Percentage Correctly Predicted (PCP) | 0.713 | 0.736 | 0.732 | 0.745 | 0.727 | 0.753 | 0.727 | 0.743 | 0.710 | 0.728 |
| Percentage Reduction in Error (PRE) | 0.234 | 0.294 | 0.283 | 0.316 | 0.267 | 0.338 | 0.267 | 0.309 | 0.224 | 0.272 |
| BIC | -69395.94 | -68942.223 | -70103.303 | -69587.507 | -69666.508 | -69305.313 | -69624.368 | -69096.806 | -70035.456 | -69586.162 |
| AIC | 1.201 | 1.129 | 1.181 | 1.109 | 1.160 | 1.094 | 1.188 | 1.119 | 1.236 | 1.151 |
| Deviance | 10529.695 | 9738.971 | 10427.467 | 9627.440 | 10158.319 | 9483.987 | 10432.350 | 9664.935 | 10969.614 | 10041.910 |
| Statistical inference |  |  |  |  |  |  |  |  |  |  |
| LR | 1721.416 | 2355.538 | 1898.80 | 2534.244 | 2064.627 | 2571.315 | 1818.553 | 2423.316 | 1417.923 | 2172.985 |
| Prob > LR | 0.0000 | 0.0000 | 0.0000 | 0.0000 | 0.0000 | 0.0000 | 0.0000 | 0.0000 | 0.0000 | 0.0000 |
| F Test | 85.43 | 108.07 | 89.92 | 113.94 | 103.06 | 165.82 | 93.89 | 114.11 | 63.50 | 98.03 |
| *P* = 0 (*p*-value) | 0.0000 | 0.0000 | 0.0000 | 0.0000 | 0.0000 | 0.0000 | 0.0000 | 0.0000 | 0.0000 | 0.0000 |

The dependent variable in columns (1) -(10) is the probability of being credit constrained for firms in stressed countries. MP_t−2_ is the one-year lag (equivalent to two survey waves) of the logarithm the assets of individual central bank balance sheets - minus autonomous factors - for stressed countries. Profit decreased, credit history deteriorated, own outlook deteriorated, and own capital deteriorated are all categorical variables which proxy firm risk from the firm’s viewpoint. Innovation is a categorical variable which proxies if the firm innovated in the previous six months and is a measure of firm risk. Country-sector fixed effects, time sector fixed effects, bank controls and macro controls (both lagged by one-year - equivalent to two survey waves) are included in all specifications. Firm controls are added in columns (2), (4), (6), (8) and (10). Robust standard errors are in parentheses. ***, **, * represent significance at the 1%, 5% and 10%, respectively
