# Supplementary material for: A methodological framework for exploring SME finance with SAFE data
Source: PLoS One. 2024 Aug 29;19(8):e0307361. doi: 10.1371/journal.pone.0307361 (PMC11361696; doi:10.1371/journal.pone.0307361)
Supplement: S5 Table — (DOCX) [file pone.0307361.s006.docx]

**S5 Table. H2, probit, Probability that the implementation of UMP leads to risky firms being less credit constrained.**

| Variables | (1) | (2) | (3) | (4) | (5) | (6) | (7) | (8) | (9) | (10) |
| --- | --- | --- | --- | --- | --- | --- | --- | --- | --- | --- |
| MP_t−2_ | -0.00733 | -0.00823 | -0.00871 | -0.00923 | -0.00458 | -0.00648 | -0.0179 | -0.0171 | -0.0159 | -0.0169 |
|  | (0.0294) | (0.0282) | (0.0284) | (0.0274) | (0.0289) | (0.0278) | (0.0287) | (0.0277) | (0.0297) | (0.0283) |
| Profit decreased | 0.0377 | 0.0657 |  |  |  |  |  |  |  |  |
|  | (0.124) | (0.120) |  |  |  |  |  |  |  |  |
| MP_t−2_ X Profit decreased | 0.0110 | 0.00521 |  |  |  |  |  |  |  |  |
|  | (0.0104) | (0.0101) |  |  |  |  |  |  |  |  |
| Credit history deteriorated |  |  | 0.105 | 0.0990 |  |  |  |  |  |  |
|  |  |  | (0.209) | (0.205) |  |  |  |  |  |  |
| MP_t-2_ x Credit history |  |  | 0.0151 | 0.0107 |  |  |  |  |  |  |
|  |  |  | (0.0174) | (0.0170) |  |  |  |  |  |  |
| Own outlook deteriorated |  |  |  |  | 0.174 | 0.114 |  |  |  |  |
|  |  |  |  |  | (0.140) | (0.135) |  |  |  |  |
| MP_t-2_ x Own outlook |  |  |  |  | 0.00654 | 0.00836 |  |  |  |  |
|  |  |  |  |  | (0.0117) | (0.0113) |  |  |  |  |
| Own capital deteriorated |  |  |  |  |  |  | -0.0434 | -0.0611 |  |  |
|  |  |  |  |  |  |  | (0.175) | (0.169) |  |  |
| MP_t-2_ x Own capital |  |  |  |  |  |  | 0.0274* | 0.0234 |  |  |
|  |  |  |  |  |  |  | (0.0148) | (0.0143) |  |  |
| Innovation |  |  |  |  |  |  |  |  | -0.119 | -0.0960 |
|  |  |  |  |  |  |  |  |  | (0.127) | (0.122) |
| MP_t-2_ x innovation |  |  |  |  |  |  |  |  | 0.0111 | 0.00955 |
| **Bank characteristic variables** |  |  |  |  |  |  |  |  |  |  |
|  |  |  |  |  |  |  |  |  | (0.0107) | (0.0103) |
| Non-performing loans_t-2_ | -0.000188 | -0.000596 | -0.00131 | -0.00132 | 0.00530** | 0.00421** | -0.00149 | -0.00173 | -0.00247 | -0.00228 |
|  | (0.00205) | (0.00197) | (0.00203) | (0.00194) | (0.00205) | (0.00197) | (0.00204) | (0.00196) | (0.00208) | (0.00198) |
| Reg tier capital ratio_t-2_ | -0.00247 | -0.00194 | -0.00183 | -0.00147 | 0.00356 | 0.00295 | -0.00342 | -0.00290 | -0.00192 | -0.00178 |
|  | (0.00635) | (0.00605) | (0.00626) | (0.00598) | (0.00624) | (0.00598) | (0.00630) | (0.00603) | (0.00645) | (0.00610) |
| **Macroeconomic variables** |  |  |  |  |  |  |  |  |  |  |
|  |  |  |  |  |  |  |  |  |  |  |
| Unemployment_t-2_ | 0.00147 | 0.00145 | 0.00184 | 0.00174 | 0.00169 | 0.00169 | 0.00169 | 0.00147 | 0.00135 | 0.00130 |
|  | (0.00167) | (0.00162) | (0.00164) | (0.00159) | (0.00164) | (0.00159) | (0.00166) | (0.00161) | (0.00169) | (0.00163) |
| Inflation_t-2_ | 0.00583 | 0.00527 | 0.0103 | 0.00832 | -0.00453 | -0.00362 | 0.0146 | 0.0118 | 0.0123 | 0.00961 |
|  | (0.0140) | (0.0135) | (0.0138) | (0.0133) | (0.0137) | (0.0133) | (0.0138) | (0.0133) | (0.0142) | (0.0137) |
| **Firm characteristic variables** |  |  |  |  |  |  |  |  |  |  |
| Micro |  | 0.0839*** |  | 0.0777*** |  | 0.0740*** |  | 0.0790*** |  | 0.0916*** |
|  |  | (0.0178) |  | (0.0175) |  | (0.0175) |  | (0.0176) |  | (0.0179) |
| Small |  | 0.00956 |  | 0.00351 |  | 0.00190 |  | 0.00822 |  | 0.00871 |
|  |  | (0.0151) |  | (0.0148) |  | (0.0148) |  | (0.0149) |  | (0.0152) |
| Trade |  | -0.0393 |  | -0.0330 |  | -0.0413 |  | -0.0305 |  | -0.0346 |
|  |  | (0.0282) |  | (0.0283) |  | (0.0280) |  | (0.0280) |  | (0.0285) |
| Industry |  | -0.0914*** |  | -0.0735*** |  | -0.0925*** |  | -0.0805*** |  | -0.0855*** |
|  |  | (0.0278) |  | (0.0278) |  | (0.0276) |  | (0.0276) |  | (0.0282) |
| Less than 2yrs |  | 0.107** |  | 0.0807 |  | 0.0894* |  | 0.0918* |  | 0.0868* |
|  |  | (0.0506) |  | (0.0507) |  | (0.0516) |  | (0.0505) |  | (0.0525) |
| Between 2-5yrs |  | 0.123*** |  | 0.129*** |  | 0.111*** |  | 0.126*** |  | 0.124*** |
|  |  | (0.0252) |  | (0.0255) |  | (0.0249) |  | (0.0254) |  | (0.0256) |
| Between 5-10yrs |  | 0.0256 |  | 0.0231 |  | 0.0167 |  | 0.0216 |  | 0.0160 |
|  |  | (0.0156) |  | (0.0156) |  | (0.0155) |  | (0.0158) |  | (0.0158) |
| Turnover up to 2mn |  | 0.271*** |  | 0.272*** |  | 0.250*** |  | 0.278*** |  | 0.295*** |
|  |  | (0.0370) |  | (0.0365) |  | (0.0367) |  | (0.0366) |  | (0.0372) |
| Turnover between 2-10mn |  | 0.155*** |  | 0.157*** |  | 0.143*** |  | 0.168*** |  | 0.171*** |
|  |  | (0.0357) |  | (0.0350) |  | (0.0353) |  | (0.0351) |  | (0.0358) |
| Turnover between 10-50mn |  | 0.0572 |  | 0.0556 |  | 0.0389 |  | 0.0634* |  | 0.0589 |
|  |  | (0.0358) |  | (0.0351) |  | (0.0354) |  | (0.0352) |  | (0.0359) |
| Stand-alone firm |  | -0.0942*** |  | -0.0912*** |  | -0.0974*** |  | -0.0905*** |  | -0.0964*** |
|  |  | (0.0212) |  | (0.0206) |  | (0.0211) |  | (0.0210) |  | (0.0214) |
| Observations | 8,836 | 8,726 | 8,896 | 8,779 | 8,826 | 8,707 | 8,849 | 8,734 | 8,943 | 8,820 |
| Country*Sector FE | Yes | Yes | Yes | Yes | Yes | Yes | Yes | Yes | Yes | Yes |
| Time FE | Yes | Yes | Yes | Yes | Yes | Yes | Yes | Yes | Yes | Yes |
| Bank Controls | Yes | Yes | Yes | Yes | Yes | Yes | Yes | Yes | Yes | Yes |
| Macro Controls | Yes | Yes | Yes | Yes | Yes | Yes | Yes | Yes | Yes | Yes |
| Other Firm Controls | No | Yes | No | Yes | No | Yes | No | Yes | No | Yes |
| Goodness of Fit |  |  |  |  |  |  |  |  |  |  |
| Mc Fadden’s Pseudo R^2^ | 0.138 | 0.194 | 0.150 | 0.206 | 0.165 | 0.217 | 0.146 | 0.199 | **0.112** | 0.177 |
| Mc Fadden’s Adjusted Pseudo R^2^ | 0.131 | 0.184 | 0.143 | 0.197 | 0.158 | 0.208 | 0.139 | 0.189 | **0.105** | 0.168 |
| Percentage Correctly Predicted (PCP) | 0.713 | 0.737 | 0.732 | 0.744 | 0.728 | 0.753 | 0.727 | 0.741 | **0.710** | 0.732 |
| Percentage Reduction in Error (PRE) | 0.234 | 0.296 | 0.282 | 0.314 | 0.269 | 0.336 | 0.268 | 0.305 | **0.223** | 0.281 |
| Expected PCP Herron | 0.615 | 0.644 | 0.623 | 0.651 | 0.630 | 0.657 | 0.620 | 0.647 | **0.601** | 0.635 |
| Expected PRE Herron | 0.178 | 0.239 | 0.194 | 0.254 | 0.210 | 0.267 | 0.187 | 0.245 | **0.147** | 0.220 |
| BIC | -69855.328 | -69381.42 | -70539.549 | -70004.790 | -70084.179 | -69489.671 | -70071.319 | -69523.431 | **-70510.508** | -7000.456 |
| AIC | 1.149 | 1.078 | 1.132 | 1.062 | 1.113 | 1.046 | 1.137 | 1.070 | **1.182** | 1.099 |
| Area under ROC | 0.7344 | 0.7786 | 0.7429 | 0.7882 | 0.7584 | 0.7966 | 0.7325 | 0.7807 | **0.6999** | 0.7642 |
| Deviance | 10070.314 | 9299.775 | 9991.221 | 9210.157 | 9740.648 | 9000.257 | 9985.4 | 9238.310 | 10494.563 | 9586.616 |
| Statistical inference |  |  |  |  |  |  |  |  |  |  |
| Wald Test *X*^2^ | 1396.27 | 1811.79 | 1458.26 | 1882.36 | 1586.68 | 1922.91 | 1390.82 | 1813.46 | 1174.57 | 1739.07 |
| Prob > Chi^2^ | 0.0000 | 0.0000 | 0.0000 | 0.0000 | 0.0000 | 0.0000 | 0.0000 | 0.0000 | 0.0000 | 0.0000 |
| LR | 1616.225 | 2237.365 | 1766.983 | 2391.042 | 1918.979 | 2499.446 | 1700.888 | 2292.815 | **1322.077** | 2065.332 |
| Prob > LR | 0.0000 | 0.0000 | 0.0000 | 0.0000 | 0.0000 | 0.0000 | 0.0000 | 0.0000 | 0.0000 | 0.0000 |

The dependent variable in columns (1) -(10) is the probability of being credit constrained for firms in stressed countries. MP_t−2_ is the one-year lag (equivalent to two survey waves) of the logarithm the assets of individual central bank balance sheets - minus autonomous factors - for stressed countries. Profit decreased, credit history deteriorated, own outlook deteriorated, and own capital deteriorated are all categorical variables which proxy firm risk from the firm’s viewpoint. Innovation is a categorical variable which proxies if the firm innovated in the previous six months and is a measure of firm risk. Country-sector fixed effects, time sector fixed effects, bank controls and macro controls (both lagged by one-year - equivalent to two survey waves) are included in all specifications. Firm controls are added in columns (2), (4), (6), (8) and (10). Robust standard errors are in parentheses. ***, **, * represent significance at the 1%, 5% and 10%, respectively*.*
