# Supplementary material for: A methodological framework for exploring SME finance with SAFE data
Source: PLoS One. 2024 Aug 29;19(8):e0307361. doi: 10.1371/journal.pone.0307361 (PMC11361696; doi:10.1371/journal.pone.0307361)
Supplement: S6 Table — (DOCX) [file pone.0307361.s007.docx]

**S6 Table. H2, logit, Probability that the implementation of UMP leads to risky firms being less credit constrained.**

| Credit constrained | (1) | (2) | (3) | (4) | (5) | (6) | (7) | (8) | (9) | (10) |
| --- | --- | --- | --- | --- | --- | --- | --- | --- | --- | --- |
| Variables |  |  |  |  |  |  |  |  |  |  |
|  |  |  |  |  |  |  |  |  |  |  |
| MP_t−2_ | -0.00921 | -0.00917 | -0.0117 | -0.00999 | -0.00734 | -0.00796 | -0.0219 | -0.0191 | -0.0177 | -0.0170 |
|  | (0.0290) | (0.0278) | (0.0277) | (0.0268) | (0.0286) | (0.0275) | (0.0282) | (0.0273) | (0.0293) | (0.0280) |
| Profit decreased | 0.0147 | 0.0429 |  |  |  |  |  |  |  |  |
|  | (0.121) | (0.117) |  |  |  |  |  |  |  |  |
| MP_t−2_ X Profit decreased | 0.0129 | 0.00707 |  |  |  |  |  |  |  |  |
|  | (0.0102) | (0.00988) |  |  |  |  |  |  |  |  |
| Credit history deteriorated |  |  | 0.164 | 0.154 |  |  |  |  |  |  |
|  |  |  | (0.208) | (0.205) |  |  |  |  |  |  |
| MP_t-2_ x Credit history |  |  | 0.00974 | 0.00641 |  |  |  |  |  |  |
|  |  |  | (0.0173) | (0.0170) |  |  |  |  |  |  |
| Own outlook deteriorated |  |  |  |  | 0.160 | 0.0941 |  |  |  |  |
|  |  |  |  |  | (0.137) | (0.133) |  |  |  |  |
| MP_t-2_ X Own outlook |  |  |  |  | 0.00735 | 0.00972 |  |  |  |  |
|  |  |  |  |  | (0.0115) | (0.0112) |  |  |  |  |
| Own capital deteriorated |  |  |  |  |  |  | 0.000610 | -0.0290 |  |  |
|  |  |  |  |  |  |  | (0.173) | (0.168) |  |  |
| MP_t-2_ x Own capital |  |  |  |  |  |  | 0.0234 | 0.0204 |  |  |
|  |  |  |  |  |  |  | (0.0146) | (0.0142) |  |  |
| Innovation |  |  |  |  |  |  |  |  | -0.114 | -0.0810 |
|  |  |  |  |  |  |  |  |  | (0.125) | (0.120) |
| MP_t-2_ X innovation |  |  |  |  |  |  |  |  | 0.0108 | 0.00829 |
|  |  |  |  |  |  |  |  |  | (0.0105) | (0.0101) |
| **Bank characteristic variables** |  |  |  |  |  |  |  |  |  |  |
| Non-performing loans_t-2_ | -0.000276 | -0.000576 | -0.00158 | -0.00145 | 0.00473** | 0.00394** | -0.00175 | -0.00179 | -0.00261 | -0.00221 |
|  | (0.00205) | (0.00196) | (0.00202) | (0.00193) | (0.00205) | (0.00196) | (0.00203) | (0.00195) | (0.00208) | (0.00197) |
| Reg tier capital ratio_t-2_ | -0.00247 | -0.00179 | -0.00253 | -0.00185 | 0.00304 | 0.00310 | -0.00383 | -0.00343 | -0.00220 | -0.00171 |
|  | (0.00635) | (0.00603) | (0.00621) | (0.00592) | (0.00626) | (0.00598) | (0.00628) | (0.00599) | (0.00648) | (0.00609) |
| **Macroeconomic variables** |  |  |  |  |  |  |  |  |  |  |
| Unemployment_t-2_ | 0.00143 | 0.00130 | 0.00182 | 0.00163 | 0.00144 | 0.00141 | 0.00154 | 0.00119 | 0.00130 | 0.00109 |
|  | (0.00167) | (0.00162) | (0.00164) | (0.00159) | (0.00164) | (0.00159) | (0.00167) | (0.00162) | (0.00169) | (0.00164) |
| Inflation_t-2_ | 0.00538 | 0.00452 | 0.00990 | 0.00740 | -0.00519 | -0.00398 | 0.0139 | 0.0108 | 0.0116 | 0.00848 |
|  | (0.0140) | (0.0135) | (0.0137) | (0.0132) | (0.0136) | (0.0132) | (0.0137) | (0.0132) | (0.0141) | (0.0136) |
| **Firm characteristic variables** |  |  |  |  |  |  |  |  |  |  |
| Micro |  | 0.0837*** |  | 0.0769*** |  | 0.0721*** |  | 0.0785*** |  | 0.0908*** |
|  |  | (0.0178) |  | (0.0176) |  | (0.0175) |  | (0.0176) |  | (0.0179) |
| Small |  | 0.0116 |  | 0.00437 |  | 0.00223 |  | 0.00907 |  | 0.00990 |
|  |  | (0.0154) |  | (0.0152) |  | (0.0151) |  | (0.0152) |  | (0.0156) |
| Trade |  | -0.0400 |  | -0.0324 |  | -0.0410 |  | -0.0301 |  | -0.0348 |
|  |  | (0.0274) |  | (0.0279) |  | (0.0275) |  | (0.0273) |  | (0.0278) |
| Industry |  | -0.0937*** |  | -0.0742*** |  | -0.0940*** |  | -0.0809*** |  | -0.0874*** |
|  |  | (0.0274) |  | (0.0277) |  | (0.0273) |  | (0.0272) |  | (0.0277) |
| Lessthan2yrs |  | 0.107** |  | 0.0795 |  | 0.0932* |  | 0.0912* |  | 0.0888* |
|  |  | (0.0480) |  | (0.0492) |  | (0.0496) |  | (0.0488) |  | (0.0508) |
| Between2and5yrs |  | 0.120*** |  | 0.127*** |  | 0.110*** |  | 0.123*** |  | 0.121*** |
|  |  | (0.0246) |  | (0.0250) |  | (0.0241) |  | (0.0249) |  | (0.0250) |
| Between5and10yrs |  | 0.0258* |  | 0.0237 |  | 0.0175 |  | 0.0214 |  | 0.0167 |
|  |  | (0.0155) |  | (0.0155) |  | (0.0153) |  | (0.0158) |  | (0.0157) |
| Turnoverupto2mn |  | 0.290*** |  | 0.289*** |  | 0.271*** |  | 0.292*** |  | 0.315*** |
|  |  | (0.0420) |  | (0.0414) |  | (0.0416) |  | (0.0414) |  | (0.0425) |
| Turnoverbetween2and10mn |  | 0.175*** |  | 0.175*** |  | 0.164*** |  | 0.183*** |  | 0.192*** |
|  |  | (0.0409) |  | (0.0403) |  | (0.0405) |  | (0.0402) |  | (0.0414) |
| Turnoverbetween10and50mn |  | 0.0698* |  | 0.0660 |  | 0.0533 |  | 0.0721* |  | 0.0717* |
|  |  | (0.0414) |  | (0.0407) |  | (0.0410) |  | (0.0407) |  | (0.0420) |
| Individual or family-owned |  | -0.0113 |  | -0.0115 |  | -0.00267 |  | -0.00854 |  | -0.00914 |
|  |  | (0.0154) |  | (0.0149) |  | (0.0151) |  | (0.0151) |  | (0.0155) |
| Stand-alone firm |  | -0.0974*** |  | -0.0935*** |  | -0.0991*** |  | -0.0918*** |  | -0.0988*** |
|  |  | (0.0219) |  | (0.0210) |  | (0.0217) |  | (0.0215) |  | (0.0221) |
| Observations | 8,836 | 8,726 | 8,896 | 8,779 | 8,826 | 8,707 | 8,849 | 8,734 | 8,943 | 8,820 |
| Country*Sector FE | Yes | Yes | Yes | Yes | Yes | Yes | Yes | Yes | Yes | Yes |
| Time FE | Yes | Yes | Yes | Yes | Yes | Yes | Yes | Yes | Yes | Yes |
| Bank Controls | Yes | Yes | Yes | Yes | Yes | Yes | Yes | Yes | Yes | Yes |
| Macro Controls | Yes | Yes | Yes | Yes | Yes | Yes | Yes | Yes | Yes | Yes |
| Other Firm Controls | No | Yes | No | Yes | No | Yes | No | Yes | No | Yes |
| Goodness of fit (LPM) |  |  |  |  |  |  |  |  |  |  |
| Mc Fadden’s Pseudo R^2^ | 0.138 | 0.195 | 0.151 | 0.2067 | 0.1648 | 0.218 | 0.146 | 0.199 | 0.11 | 0.178 |
| Mc Fadden’s Adjusted Pseudo R2 | 0.132 | 0.185 | 0.144 | 0.197 | 0.158 | 0.209 | 0.139 | 0.190 | 0.10 | 0.168 |
| Percentage Correctly Predicted (PCP) | 0.714 | 0.737 | 0.732 | 0.744 | 0.728 | 0.753 | 0.727 | 0.743 | 0.710 | 0.732 |
| Percentage Reduction in Error (PRE) | 0.235 | 0.298 | 0.282 | 0.315 | 0.269 | 0.338 | 0.267 | 0.308 | 0.223 | 0.281 |
| Expected PCP Herron | 0.615 | 0.645 | 0.623 | 0.652 | 0.631 | 0.658 | 0.620 | 0.648 | 0.601 | 0.636 |
| Expected PRE Herron | 0.178 | 0.241 | 0.194 | 0.256 | 0.210 | 0.270 | 0.188 | 0.247 | 0.147 | 0.222 |
| BIC | -69856.429 | -69389.565 | -70542.183 | -70011.877 | -70086.919 | -69500.148 | -70073.296 | -69528.432 | -70511.273 | -70047.971 |
| AIC | 1.149 | 1.077 | 1.132 | 1.061 | 1.112 | 1.045 | 1.137 | 1.070 | 1.182 | 1.099 |
| Area under the ROC curve | 0.7344 | 0.7791 | 0.7429 | 0.7884 | 0.7586 | 0.7969 | 0.7327 | 0.7809 | 0.6999 | 0.7649 |
| Deviance | 10069.213 | 9291.57 | 9988.587 | 9303.071 | 9737.908 | 8989.851 | 9983.423 | 9233.308 | 10493.798 | 9580.101 |
| Statistical inference |  |  |  |  |  |  |  |  |  |  |
| Wald Test *X*^2^ | 1245.88 | 1568.64 | 1331.69 | 1646.48 | 1394.37 | 1660.99 | 1253.02 | 1573.64 | 1058.39 | 1508.64 |
| Prob > Chi^2^ | 0.00 | 0.00 | 0.00 | 0.00 | 0.00 | 0.00 | 0.00 | 0.00 | 0.00 | 0.00 |
| LR (higher better) | 1617.325 | 2245.510 | 1769.618 | 2398.129 | 1921.719 | 2509.923 | 1702.865 | 2297.816 | 1322.842 | 2071.848 |
| Prob > LR | 0.00 | 0.00 | 0.00 | 0.00 | 0.00 | 0.00 | 0.00 | 0.00 | 0.00 | 0.00 |

The dependent variable in columns (1) -(10) is the probability of being credit constrained for firms in stressed countries. Reported estimates are conditional marginal effects drawn from logit regression models with sample selection for the pooled sample of SMEs. MP_t−2_ is the one-year lag (equivalent to two survey waves) of the logarithm the assets of individual central bank balance sheets - minus autonomous factors - for stressed countries. Profit decreased, credit history deteriorated, own outlook deteriorated, and own capital deteriorated are all categorical variables which proxy firm risk from the firm’s viewpoint. Innovation is a categorical variable which proxies if the firm innovated in the previous six months and is a measure of firm risk. Country-sector fixed effects, time sector fixed effects, bank controls and macro controls (both lagged by one-year - equivalent to two survey waves) are included in all specifications. Firm controls are added in columns (2), (4), (6), (8) and (10). Robust standard errors are in parentheses. ***, **, * represent significance at the 1%, 5% and 10%, respectively.
