# Supplementary material for: A methodological framework for exploring SME finance with SAFE data
Source: PLoS One. 2024 Aug 29;19(8):e0307361. doi: 10.1371/journal.pone.0307361 (PMC11361696; doi:10.1371/journal.pone.0307361)
Supplement: S7 Table — (DOCX) [file pone.0307361.s008.docx]

**S7 Table. Correlation tables**

**Table 1. Matrix of correlations H1: MP_t-2_ X Debt to assets increased**

| Variables | (1) | | (2) | (3) | (4) | (5) | (6) | (7) | (8) | (9) | (10) | (11) | (12) | (13) | (14) | (15) | (16) | (17) | (18) | (19) |
| --- | --- | --- | --- | --- | --- | --- | --- | --- | --- | --- | --- | --- | --- | --- | --- | --- | --- | --- | --- | --- |
| (1) MP_t-2_ | 1.00 | |  |  |  |  |  |  |  |  |  |  |  |  |  |  |  |  |  |  |
| (2) Debt to assets increased | -0.02 | | 1.00 |  |  |  |  |  |  |  |  |  |  |  |  |  |  |  |  |  |
| (3) MP_t-2_ X Debt to assets increased | 0.03 | | 1.00 | 1.00 |  |  |  |  |  |  |  |  |  |  |  |  |  |  |  |  |
| (4) Non-performing loans_t_ **_-2_** | -0.06 | | -0.01 | -0.01 | 1.00 |  |  |  |  |  |  |  |  |  |  |  |  |  |  |  |
| (5) Tier 1 Capital Ratio_t_ **_-2_** | -0.69 | | 0.03 | -0.00 | -0.11 | 1.00 |  |  |  |  |  |  |  |  |  |  |  |  |  |  |
| (6) Unemployment_t-2_ | -0.02 | | 0.03 | 0.03 | 0.44 | 0.15 | 1.00 |  |  |  |  |  |  |  |  |  |  |  |  |  |
| (7) Inflation_t-2_ | 0.17 | | -0.01 | -0.01 | -0.31 | -0.03 | -0.42 | 1.00 |  |  |  |  |  |  |  |  |  |  |  |  |
| (8) Micro | -0.08 | | 0.01 | 0.00 | 0.13 | 0.08 | 0.14 | -0.09 | 1.00 |  |  |  |  |  |  |  |  |  |  |  |
| (9) Small | -0.01 | | -0.00 | -0.01 | -0.03 | 0.01 | -0.02 | 0.02 | -0.44 | 1.00 |  |  |  |  |  |  |  |  |  |  |
| (10) Trade | -0.03 | | 0.01 | 0.01 | -0.01 | 0.01 | -0.00 | 0.01 | 0.15 | 0.05 | 1.00 |  |  |  |  |  |  |  |  |  |
| (11) Industry | -0.00 | | -0.01 | -0.01 | 0.05 | 0.01 | 0.06 | -0.05 | -0.04 | 0.06 | -0.66 | 1.00 |  |  |  |  |  |  |  |  |
| (12) Less than 2 years | 0.01 | | 0.01 | 0.01 | -0.02 | 0.00 | -0.02 | 0.02 | 0.06 | -0.02 | 0.02 | 0.00 | 1.00 |  |  |  |  |  |  |  |
| (13) Between 2 and 5 years | -0.01 | | 0.02 | 0.02 | 0.01 | -0.01 | -0.00 | -0.00 | 0.08 | -0.00 | 0.04 | -0.01 | -0.02 | 1.00 |  |  |  |  |  |  |
| (14) Between 5 and 10 years | -0.02 | | 0.02 | 0.02 | 0.00 | -0.00 | 0.00 | -0.02 | 0.13 | -0.01 | 0.07 | -0.02 | -0.03 | -0.05 | 1.00 |  |  |  |  |  |
| (15) Turnover up to 2mn | -0.09 | | 0.02 | 0.01 | 0.13 | 0.06 | 0.14 | -0.08 | 0.67 | -0.01 | 0.25 | -0.12 | 0.06 | 0.09 | 0.12 | 1.00 |  |  |  |  |
| (16) Turnover 2-10mn | 0.01 | | -0.01 | -0.01 | -0.03 | -0.01 | -0.03 | 0.02 | -0.28 | 0.34 | 0.02 | 0.08 | -0.02 | -0.02 | -0.03 | -0.49 | 1.00 |  |  |  |
| (17) Turnover 10-50mn | 0.05 | | -0.01 | -0.00 | -0.07 | -0.03 | -0.06 | 0.04 | -0.33 | -0.18 | -0.17 | 0.17 | -0.03 | -0.05 | -0.07 | -0.44 | -0.31 | 1.00 |  |  |
| (18) Individual or family owned | -0.04 | | -0.02 | -0.02 | 0.10 | -0.01 | 0.03 | -0.03 | 0.20 | 0.07 | 0.04 | 0.05 | -0.00 | 0.00 | 0.01 | 0.23 | 0.03 | -0.14 | 1.00 |  |
| (19) Stand-alone firm | -0.03 | | -0.01 | -0.01 | 0.11 | 0.00 | 0.09 | -0.05 | 0.18 | 0.07 | 0.07 | 0.03 | -0.01 | 0.01 | 0.00 | 0.22 | 0.04 | -0.14 | 0.33 | 1.00 |
|  |  |  |  |  |  |  |  |  |  |  |  |  |  |  |  |  |  |  |  |  |

Definitions and sources of all the variables are reported in Table 3.4 in Chapter 3. In terms of categorical variables, the omitted variable - which also serves as the reference category - for firm size is medium (50 to <250); the omitted firm turnover is above €50mn; the omitted sector is construction and the omitted age is more than 10 years.

**Table 2. Matrix of correlations H2: MP_t-2_ X Profit Decreased**

| Variables | (1) | (2) | (3) | (4) | (5) | (6) | (7) | (8) | (9) | (10) | (11) | (12) | (13) | (14) | (15) | (16) | (17) | (18) | (19) |
| --- | --- | --- | --- | --- | --- | --- | --- | --- | --- | --- | --- | --- | --- | --- | --- | --- | --- | --- | --- |
| (1) MP_t-2_ | 1.00 |  |  |  |  |  |  |  |  |  |  |  |  |  |  |  |  |  |  |
| (2) Profit decreased | -0.02 | 1.00 |  |  |  |  |  |  |  |  |  |  |  |  |  |  |  |  |  |
| (3) MP_t-2_ X Profit decreased | 0.04 | 0.99 | 1.00 |  |  |  |  |  |  |  |  |  |  |  |  |  |  |  |  |
| (4) Non-performing loans_t_ **_-2_** | -0.06 | 0.11 | 0.10 | 1.00 |  |  |  |  |  |  |  |  |  |  |  |  |  |  |  |
| (5) Tier 1 Capital Ratio_t_ **_-2_** | -0.69 | 0.03 | -0.01 | -0.11 | 1.00 |  |  |  |  |  |  |  |  |  |  |  |  |  |  |
| (6) Unemployment_t-2_ | -0.03 | 0.09 | 0.09 | 0.44 | 0.15 | 1.00 |  |  |  |  |  |  |  |  |  |  |  |  |  |
| (7) Inflation_t-2_ | 0.17 | -0.06 | -0.04 | -0.31 | -0.03 | -0.42 | 1.00 |  |  |  |  |  |  |  |  |  |  |  |  |
| (8) Micro | -0.08 | 0.12 | 0.11 | 0.14 | 0.08 | 0.15 | -0.09 | 1.00 |  |  |  |  |  |  |  |  |  |  |  |
| (9) Small | -0.01 | -0.01 | -0.01 | -0.04 | 0.00 | -0.02 | 0.02 | -0.44 | 1.00 |  |  |  |  |  |  |  |  |  |  |
| (10) Trade | -0.03 | 0.00 | 0.00 | -0.01 | 0.01 | -0.01 | 0.01 | 0.15 | 0.05 | 1.00 |  |  |  |  |  |  |  |  |  |
| (11) Industry | -0.01 | 0.02 | 0.02 | 0.05 | 0.01 | 0.06 | -0.05 | -0.04 | 0.06 | -0.66 | 1.00 |  |  |  |  |  |  |  |  |
| (12) Less than 2 years | 0.01 | -0.01 | -0.01 | -0.02 | -0.00 | -0.02 | 0.02 | 0.06 | -0.01 | 0.02 | 0.00 | 1.00 |  |  |  |  |  |  |  |
| (13) Between 2 and 5 years | -0.01 | 0.01 | 0.01 | 0.01 | -0.00 | 0.00 | -0.01 | 0.08 | -0.00 | 0.04 | -0.01 | -0.02 | 1.00 |  |  |  |  |  |  |
| (14) Between 5 and 10 years | -0.02 | -0.01 | -0.01 | 0.00 | -0.00 | -0.00 | -0.02 | 0.13 | -0.01 | 0.07 | -0.03 | -0.03 | -0.05 | 1.00 |  |  |  |  |  |
| (15) Turnover up to 2mn | -0.08 | 0.12 | 0.12 | 0.13 | 0.06 | 0.14 | -0.08 | 0.67 | -0.01 | 0.26 | -0.12 | 0.05 | 0.09 | 0.12 | 1.00 |  |  |  |  |
| (16) Turnover 2-10mn | 0.01 | -0.02 | -0.02 | -0.03 | -0.01 | -0.03 | 0.02 | -0.28 | 0.34 | 0.02 | 0.08 | -0.02 | -0.02 | -0.03 | -0.49 | 1.00 |  |  |  |
| (17) Turnover 10-50mn | 0.05 | -0.06 | -0.06 | -0.07 | -0.04 | -0.06 | 0.04 | -0.33 | -0.18 | -0.17 | 0.17 | -0.03 | -0.05 | -0.07 | -0.44 | -0.31 | 1.00 |  |  |
| (18) Individ or family owned | -0.04 | 0.04 | 0.03 | 0.10 | -0.01 | 0.03 | -0.04 | 0.20 | 0.07 | 0.04 | 0.05 | -0.00 | -0.00 | 0.01 | 0.23 | 0.03 | -0.14 | 1.00 |  |
| (19) Stand-alone firm | -0.03 | 0.03 | 0.02 | 0.11 | 0.00 | 0.09 | -0.05 | 0.18 | 0.07 | 0.07 | 0.03 | -0.01 | 0.01 | 0.00 | 0.22 | 0.04 | -0.14 | 0.33 | 1.00 |
|  |  |  |  |  |  |  |  |  |  |  |  |  |  |  |  |  |  |  |  |

Definitions and sources of all the variables are reported in Table 3.4 in Chapter 3. The omitted category for the categorical variables - which also serves as the reference category - for firm size is medium (50 to <250); the omitted firm turnover is above €50mn; the omitted sector is construction and the omitted age is more than 10 years.

**Table 3. Matrix of correlations H2: MP_t-2_ X Credit History Deteriorated**

| Variables | (1) | (2) | (3) | (4) | (5) | (6) | (7) | (8) | (9) | (10) | (11) | (12) | (13) | (14) | (15) | (16) | (17) | (18) | (19) |
| --- | --- | --- | --- | --- | --- | --- | --- | --- | --- | --- | --- | --- | --- | --- | --- | --- | --- | --- | --- |
| (1) MP_t-2_ | 1.00 |  |  |  |  |  |  |  |  |  |  |  |  |  |  |  |  |  |  |
| (2) Credit history deteriorated | -0.01 | 1.00 |  |  |  |  |  |  |  |  |  |  |  |  |  |  |  |  |  |
| (3) MP_t-2_ X Credit history | 0.02 | 1.00 | 1.00 |  |  |  |  |  |  |  |  |  |  |  |  |  |  |  |  |
| (4) Non-performing loans_t_ **_-2_** | -0.06 | 0.03 | 0.02 | 1.00 |  |  |  |  |  |  |  |  |  |  |  |  |  |  |  |
| (5) Tier 1 Capital Ratio_t_ **_-2_** | -0.69 | -0.01 | -0.02 | -0.11 | 1.00 |  |  |  |  |  |  |  |  |  |  |  |  |  |  |
| (6) Unemployment_t-2_ | -0.03 | 0.03 | 0.03 | 0.44 | 0.15 | 1.00 |  |  |  |  |  |  |  |  |  |  |  |  |  |
| (7) Inflation_t-2_ | 0.17 | -0.04 | -0.04 | -0.31 | -0.03 | -0.42 | 1.00 |  |  |  |  |  |  |  |  |  |  |  |  |
| (8) Micro | -0.08 | 0.11 | 0.11 | 0.14 | 0.08 | 0.14 | -0.09 | 1.00 |  |  |  |  |  |  |  |  |  |  |  |
| (9) Small | -0.01 | 0.00 | 0.00 | -0.04 | 0.01 | -0.02 | 0.02 | -0.44 | 1.00 |  |  |  |  |  |  |  |  |  |  |
| (10) Trade | -0.03 | 0.03 | 0.03 | -0.01 | 0.01 | -0.00 | 0.01 | 0.15 | 0.05 | 1.00 |  |  |  |  |  |  |  |  |  |
| (11) Industry | -0.00 | -0.00 | -0.00 | 0.05 | 0.01 | 0.06 | -0.05 | -0.04 | 0.06 | -0.65 | 1.00 |  |  |  |  |  |  |  |  |
| (12) Less than 2 years | 0.01 | 0.02 | 0.02 | -0.02 | 0.00 | -0.02 | 0.02 | 0.06 | -0.02 | 0.02 | 0.00 | 1.00 |  |  |  |  |  |  |  |
| (13) Between 2 and 5 years | -0.01 | 0.01 | 0.01 | 0.01 | -0.01 | -0.00 | -0.01 | 0.08 | -0.00 | 0.04 | -0.01 | -0.02 | 1.00 |  |  |  |  |  |  |
| (14) Between 5 and 10 years | -0.02 | 0.01 | 0.01 | 0.00 | -0.00 | -0.00 | -0.02 | 0.13 | -0.01 | 0.07 | -0.03 | -0.03 | -0.05 | 1.00 |  |  |  |  |  |
| (15) Turnover up to 2mn | -0.09 | 0.12 | 0.12 | 0.13 | 0.06 | 0.14 | -0.08 | 0.67 | -0.01 | 0.26 | -0.12 | 0.06 | 0.09 | 0.12 | 1.00 |  |  |  |  |
| (16) Turnover 2-10mn | 0.01 | -0.02 | -0.02 | -0.03 | -0.01 | -0.03 | 0.02 | -0.28 | 0.34 | 0.02 | 0.08 | -0.02 | -0.02 | -0.03 | -0.49 | 1.00 |  |  |  |
| (17) Turnover 10-50mn | 0.05 | -0.06 | -0.06 | -0.07 | -0.03 | -0.06 | 0.04 | -0.33 | -0.18 | -0.17 | 0.17 | -0.03 | -0.05 | -0.07 | -0.44 | -0.31 | 1.00 |  |  |
| (18) Individual or family owned | -0.04 | 0.03 | 0.03 | 0.10 | -0.01 | 0.03 | -0.04 | 0.20 | 0.06 | 0.04 | 0.05 | -0.00 | -0.00 | 0.01 | 0.23 | 0.03 | -0.14 | 1.00 |  |
| (19) Stand-alone firm | -0.03 | 0.02 | 0.02 | 0.11 | 0.00 | 0.09 | -0.05 | 0.18 | 0.07 | 0.07 | 0.03 | -0.01 | 0.01 | 0.00 | 0.22 | 0.04 | -0.14 | 0.32 | 1.00 |
|  |  |  |  |  |  |  |  |  |  |  |  |  |  |  |  |  |  |  |  |

Definitions and sources of all the variables are reported in Table 3.4 in Chapter 3. The omitted category for the categorical variables - which also serves as the reference category - for firm size is medium (50 to <250); the omitted firm turnover is above €50mn; the omitted sector is construction and the omitted age is more than 10 years.

**Table 4. Matrix of correlations H2: MP_t-2_ X Own outlook deteriorated**

| Variables | (1) | (2) | (3) | (4) | (5) | (6) | (7) | (8) | (9) | (10) | (11) | (12) | (13) | (14) | (15) | (16) | (17) | (18) | (19) |
| --- | --- | --- | --- | --- | --- | --- | --- | --- | --- | --- | --- | --- | --- | --- | --- | --- | --- | --- | --- |
| (1) MP_t-2_ | 1.00 |  |  |  |  |  |  |  |  |  |  |  |  |  |  |  |  |  |  |
| (2) Own outlook deteriorated | -0.05 | 1.00 |  |  |  |  |  |  |  |  |  |  |  |  |  |  |  |  |  |
| (3) MP_t-2_ X Own outlook | -0.00 | 1.00 | 1.00 |  |  |  |  |  |  |  |  |  |  |  |  |  |  |  |  |
| (4) Non-performing loans_t_ **_-2_** | -0.06 | 0.14 | 0.13 | 1.00 |  |  |  |  |  |  |  |  |  |  |  |  |  |  |  |
| (5) Tier 1 Capital Ratio_t_ **_-2_** | -0.69 | 0.03 | -0.00 | -0.11 | 1.00 |  |  |  |  |  |  |  |  |  |  |  |  |  |  |
| (6) Unemployment_t-2_ | -0.02 | 0.14 | 0.13 | 0.44 | 0.15 | 1.00 |  |  |  |  |  |  |  |  |  |  |  |  |  |
| (7) Inflation_t-2_ | 0.17 | -0.07 | -0.06 | -0.31 | -0.03 | -0.42 | 1.00 |  |  |  |  |  |  |  |  |  |  |  |  |
| (8) Micro | -0.08 | 0.15 | 0.15 | 0.14 | 0.08 | 0.14 | -0.09 | 1.00 |  |  |  |  |  |  |  |  |  |  |  |
| (9) Small | -0.01 | -0.01 | -0.01 | -0.04 | 0.01 | -0.02 | 0.02 | -0.44 | 1.00 |  |  |  |  |  |  |  |  |  |  |
| (10) Trade | -0.03 | 0.02 | 0.02 | -0.01 | 0.01 | -0.00 | 0.01 | 0.15 | 0.05 | 1.00 |  |  |  |  |  |  |  |  |  |
| (11) Industry | -0.00 | 0.02 | 0.02 | 0.05 | 0.01 | 0.06 | -0.05 | -0.04 | 0.06 | -0.65 | 1.00 |  |  |  |  |  |  |  |  |
| (12) Less than 2 years | 0.01 | 0.00 | 0.00 | -0.02 | 0.00 | -0.02 | 0.02 | 0.06 | -0.02 | 0.03 | 0.00 | 1.00 |  |  |  |  |  |  |  |
| (13) Between 2 and 5 years | -0.01 | 0.02 | 0.02 | 0.01 | -0.00 | -0.00 | -0.01 | 0.08 | -0.00 | 0.04 | -0.01 | -0.02 | 1.00 |  |  |  |  |  |  |
| (14) Between 5 and 10 years | -0.02 | 0.02 | 0.02 | 0.00 | -0.00 | 0.00 | -0.02 | 0.12 | -0.01 | 0.07 | -0.03 | -0.03 | -0.05 | 1.00 |  |  |  |  |  |
| (15) Turnover up to 2mn | -0.08 | 0.15 | 0.14 | 0.13 | 0.06 | 0.14 | -0.08 | 0.67 | -0.01 | 0.26 | -0.12 | 0.06 | 0.09 | 0.12 | 1.00 |  |  |  |  |
| (16) Turnover 2-10mn | 0.01 | -0.04 | -0.04 | -0.03 | -0.01 | -0.03 | 0.01 | -0.28 | 0.34 | 0.02 | 0.08 | -0.02 | -0.02 | -0.03 | -0.49 | 1.00 |  |  |  |
| (17) Turnover 10-50mn | 0.05 | -0.08 | -0.08 | -0.07 | -0.03 | -0.06 | 0.04 | -0.33 | -0.18 | -0.17 | 0.17 | -0.03 | -0.05 | -0.07 | -0.44 | -0.31 | 1.00 |  |  |
| (18) Individual or family owned | -0.04 | 0.05 | 0.04 | 0.09 | -0.01 | 0.03 | -0.04 | 0.20 | 0.07 | 0.04 | 0.05 | -0.00 | -0.00 | 0.01 | 0.23 | 0.03 | -0.14 | 1.00 |  |
| (19) Stand-alone firm | -0.03 | 0.06 | 0.05 | 0.11 | 0.00 | 0.09 | -0.05 | 0.18 | 0.07 | 0.07 | 0.03 | -0.01 | 0.01 | -0.00 | 0.22 | 0.04 | -0.14 | 0.32 | 1.00 |
|  |  |  |  |  |  |  |  |  |  |  |  |  |  |  |  |  |  |  |  |

Definitions and sources of all the variables are reported in Table 3.4 in Chapter 3. In terms of categorical variables, the omitted variable - which also serves as the reference category - for firm size is medium (50 to <250); the omitted firm turnover is above €50mn; the omitted sector is construction and the omitted age is more than 10 years.

**Table 5. Matrix of correlations H2: MP_t-2_ X Own capital deteriorated**

| Variables |  | (1) | (2) | (3) | (4) | (5) | (6) | (7) | (8) | (9) | (10) | (11) | (12) | (13) | (14) | (15) | (16) | (17) | (18) | (19) |
| --- | --- | --- | --- | --- | --- | --- | --- | --- | --- | --- | --- | --- | --- | --- | --- | --- | --- | --- | --- | --- |
| (1) MP_t-2_ |  | 1.00 |  |  |  |  |  |  |  |  |  |  |  |  |  |  |  |  |  |  |
| (2) Own capital deteriorated |  | -0.05 | 1.00 |  |  |  |  |  |  |  |  |  |  |  |  |  |  |  |  |  |
| (3) MP_t-2_ X Own capital |  | -0.01 | 1.00 | 1.00 |  |  |  |  |  |  |  |  |  |  |  |  |  |  |  |  |
| (4) Non-performing loans_t_ **_-2_** |  | -0.06 | 0.10 | 0.09 | 1.00 |  |  |  |  |  |  |  |  |  |  |  |  |  |  |  |
| (5) Tier 1 Capital Ratio_t_ **_-2_** |  | -0.69 | 0.03 | 0.01 | -0.11 | 1.00 |  |  |  |  |  |  |  |  |  |  |  |  |  |  |
| (6) Unemployment_t-2_ |  | -0.02 | 0.03 | 0.02 | 0.44 | 0.15 | 1.00 |  |  |  |  |  |  |  |  |  |  |  |  |  |
| (7) Inflation_t-2_ |  | 0.17 | -0.07 | -0.06 | -0.31 | -0.03 | -0.42 | 1.00 |  |  |  |  |  |  |  |  |  |  |  |  |
| (8) Micro |  | -0.08 | 0.16 | 0.15 | 0.14 | 0.08 | 0.14 | -0.09 | 1.00 |  |  |  |  |  |  |  |  |  |  |  |
| (9) Small |  | -0.01 | -0.03 | -0.03 | -0.04 | 0.01 | -0.02 | 0.02 | -0.44 | 1.00 |  |  |  |  |  |  |  |  |  |  |
| (10) Trade |  | -0.03 | 0.03 | 0.03 | -0.01 | 0.01 | -0.00 | 0.01 | 0.15 | 0.05 | 1.00 |  |  |  |  |  |  |  |  |  |
| (11) Industry |  | -0.00 | -0.00 | -0.00 | 0.05 | 0.01 | 0.06 | -0.05 | -0.04 | 0.06 | -0.65 | 1.00 |  |  |  |  |  |  |  |  |
| (12) Less than 2 years |  | 0.01 | 0.01 | 0.01 | -0.02 | 0.00 | -0.02 | 0.02 | 0.06 | -0.02 | 0.02 | 0.00 | 1.00 |  |  |  |  |  |  |  |
| (13) Between 2 and 5 years |  | -0.01 | 0.02 | 0.02 | 0.01 | -0.01 | -0.00 | -0.01 | 0.08 | -0.01 | 0.04 | -0.01 | -0.02 | 1.00 |  |  |  |  |  |  |
| (14) Between 5 and 10 years |  | -0.02 | 0.02 | 0.02 | 0.00 | -0.00 | -0.00 | -0.02 | 0.13 | -0.01 | 0.07 | -0.03 | -0.03 | -0.05 | 1.00 |  |  |  |  |  |
| (15) Turnover up to 2mn |  | -0.09 | 0.15 | 0.15 | 0.13 | 0.06 | 0.14 | -0.08 | 0.67 | -0.01 | 0.26 | -0.12 | 0.06 | 0.09 | 0.12 | 1.00 |  |  |  |  |
| (16) Turnover 2-10mn |  | 0.01 | -0.04 | -0.04 | -0.03 | -0.01 | -0.03 | 0.02 | -0.28 | 0.34 | 0.02 | 0.08 | -0.02 | -0.02 | -0.03 | -0.49 | 1.00 |  |  |  |
| (17) Turnover 10-50mn |  | 0.05 | -0.08 | -0.08 | -0.07 | -0.03 | -0.06 | 0.04 | -0.33 | -0.18 | -0.17 | 0.17 | -0.03 | -0.05 | -0.07 | -0.44 | -0.31 | 1.00 |  |  |
| (18) Individual or family owned |  | -0.04 | 0.03 | 0.03 | 0.10 | -0.01 | 0.03 | -0.03 | 0.20 | 0.07 | 0.04 | 0.05 | -0.00 | 0.00 | 0.01 | 0.23 | 0.03 | -0.14 | 1.00 |  |
| (19) Stand-alone firm |  | -0.03 | 0.02 | 0.02 | 0.11 | 0.00 | 0.09 | -0.05 | 0.18 | 0.07 | 0.07 | 0.03 | -0.01 | 0.01 | 0.00 | 0.22 | 0.04 | -0.14 | 0.33 | 1.00 |

Definitions and sources of all the variables are reported in Table 3.4 in Chapter 3. In terms of categorical variables, the omitted variable - which also serves as the reference category - for firm size is medium (50 to <250); the omitted firm turnover is above €50mn; the omitted sector is construction and the omitted age is more than 10 years.

**Table 6. Matrix of correlations H2: MP_t-2_ X Innovation**

| Variables | (1) | (2) | (3) | (4) | (5) | (6) | (7) | (8) | (9) | (10) | (11) | (12) | (13) | (14) | (15) | (16) | (17) | (18) | (19) |
| --- | --- | --- | --- | --- | --- | --- | --- | --- | --- | --- | --- | --- | --- | --- | --- | --- | --- | --- | --- |
| (1) MP_t-2_ | 1.00 |  |  |  |  |  |  |  |  |  |  |  |  |  |  |  |  |  |  |
| (2) innovation | 0.01 | 1.00 |  |  |  |  |  |  |  |  |  |  |  |  |  |  |  |  |  |
| (3) MP_t-2_ X Innovation | 0.07 | 0.99 | 1.00 |  |  |  |  |  |  |  |  |  |  |  |  |  |  |  |  |
| (4) Non-performing loans_t_ **_-2_** | -0.06 | -0.02 | -0.02 | 1.00 |  |  |  |  |  |  |  |  |  |  |  |  |  |  |  |
| (5) Tier 1 Capital Ratio_t_ **_-2_** | -0.69 | -0.02 | -0.06 | -0.11 | 1.00 |  |  |  |  |  |  |  |  |  |  |  |  |  |  |
| (6) Unemployment_t-2_ | -0.03 | 0.01 | 0.01 | 0.44 | 0.15 | 1.00 |  |  |  |  |  |  |  |  |  |  |  |  |  |
| (7) Inflation_t-2_ | 0.16 | 0.04 | 0.06 | -0.3 | -0.03 | -0.42 | 1.00 |  |  |  |  |  |  |  |  |  |  |  |  |
| (8) Micro | -0.08 | -0.03 | -0.03 | 0.13 | 0.08 | 0.14 | -0.09 | 1.00 |  |  |  |  |  |  |  |  |  |  |  |
| (9) Small | -0.01 | -0.01 | -0.01 | -0.04 | 0.01 | -0.02 | 0.02 | -0.44 | 1.00 |  |  |  |  |  |  |  |  |  |  |
| (10) Trade | -0.03 | -0.03 | -0.03 | -0.01 | 0.01 | -0.00 | 0.01 | 0.15 | 0.05 | 1.00 |  |  |  |  |  |  |  |  |  |
| (11) Industry | -0.00 | 0.04 | 0.04 | 0.05 | 0.01 | 0.06 | -0.05 | -0.04 | 0.06 | -0.66 | 1.00 |  |  |  |  |  |  |  |  |
| (12) Less than 2 years | 0.01 | 0.02 | 0.02 | -0.02 | 0.00 | -0.02 | 0.02 | 0.06 | -0.02 | 0.02 | 0.00 | 1.00 |  |  |  |  |  |  |  |
| (13) Between 2 and 5 years | -0.01 | 0.01 | 0.01 | 0.01 | -0.01 | -0.00 | -0.01 | 0.08 | -0.00 | 0.04 | -0.01 | -0.02 | 1.00 |  |  |  |  |  |  |
| (14) Between 5 and 10 years | -0.02 | 0.02 | 0.02 | 0.00 | -0.00 | -0.00 | -0.02 | 0.13 | -0.01 | 0.07 | -0.03 | -0.03 | -0.05 | 1.00 |  |  |  |  |  |
| (15) Turnover up to 2mn | -0.08 | -0.02 | -0.02 | 0.13 | 0.06 | 0.14 | -0.08 | 0.67 | -0.01 | 0.26 | -0.12 | 0.06 | 0.09 | 0.12 | 1.00 |  |  |  |  |
| (16) Turnover 2-10mn | 0.01 | -0.00 | -0.00 | -0.03 | -0.01 | -0.03 | 0.02 | -0.28 | 0.34 | 0.02 | 0.08 | -0.02 | -0.02 | -0.03 | -0.49 | 1.00 |  |  |  |
| (17) Turnover 10-50mn | 0.05 | 0.01 | 0.02 | -0.07 | -0.03 | -0.06 | 0.04 | -0.33 | -0.18 | -0.17 | 0.17 | -0.03 | -0.05 | -0.07 | -0.44 | -0.31 | 1.00 |  |  |
| (18) Individual or family owned | -0.04 | -0.00 | -0.01 | 0.09 | -0.01 | 0.03 | -0.03 | 0.20 | 0.06 | 0.04 | 0.05 | -0.00 | -0.00 | 0.01 | 0.23 | 0.03 | -0.14 | 1.00 |  |
| (19) Stand-alone firm | -0.03 | -0.00 | -0.01 | 0.11 | 0.00 | 0.09 | -0.05 | 0.18 | 0.07 | 0.07 | 0.03 | -0.01 | 0.01 | 0.00 | 0.22 | 0.04 | -0.14 | 0.33 | 1.00 |
|  | | | | | | | | | | | | | | | | | | | |

Definitions and sources of all the variables are reported in Table 3.4 in Chapter 3. In terms of categorical variables, the omitted variable - which also serves as the reference category - for firm size is medium (50 to <250); the omitted firm turnover is above €50mn; the omitted sector is construction and the omitted age is more than 10 years
