# Supplementary material for: A methodological framework for exploring SME finance with SAFE data
Source: PLoS One. 2024 Aug 29;19(8):e0307361. doi: 10.1371/journal.pone.0307361 (PMC11361696; doi:10.1371/journal.pone.0307361)
Supplement: Attachment — Submitted filename: Response to reviewers.docx [file pone.0307361.s009.docx]

# Response to Reviewers

Dear Reviewers,

Thank you very much for your insightful and valuable comments regarding this research paper. We have carefully addressed each comment and include a detailed response to each one below.

Reviewer#1
Dear Reviewer #1, thank you very much for your insightful comments. The research paper has been subject to a comprehensive review to ensure that all your comments are addressed. Your suggestions and recommendations makes the paper more readable and stronger. We respond to each comment in blue.

- Please write your own hypotheses and refer to the literature. Also express the hypotheses verbally.

We have updated section 4.1 Empirical model to write the hypothesis verbally and refer to the literature as follows:

For H1, $\gamma^{'}$ is an interaction term and is the main coefficient of interest that captures if firms with increased debt-to-assets decreased their probability of being credit-constrained during UMP. It is expected that UMP for leveraged firms should make accessing bank finance easier via their improved balance sheets and collateral, and this should translate into a reduction in credit constraints (80, 81). A negative relationship is, therefore, expected between the probability that a firm is credit-constrained and the interaction between increased debt-to-assets ratio and UMP.

For H2, the interaction between monetary policy and firm risk variables is the main coefficient of interest - $\gamma^{'}$ - as it captures the probability of a risky firm being credit-constrained during periods of UMP. ${FirmRisk}_{i,c,t}$is modelled categorically, and the measures for firm risk include a future predictor of risk - profit decreased in the previous six months, as well as a selection of subjective measures of risk - the firm's own view if there has been deterioration in credit history, own economic outlook and own capital in the previous six months and finally, an activity-based measure of risk - innovative activity given that such activity is more uncertain and therefore riskier. Given the low-interest rate environment generated by UMP, banks are expected to chase higher yields. This will manifest in lending to riskier firms via the monetary policy risk-taking channel (76, 82). A negative relationship is, therefore, expected between the probability that a firm is credit-constrained and the interaction between increased firm risk and UMP.

- Write clearly how this study differs from the literature.

We added the following to the introduction

This paper contributes to existing literature by introducing a methodological framework aimed at aiding researchers in determining the preferable categorical dependent model when utilising SAFE data.

- Write the table names formally. You can add hypothesis tests as notes under the table.

We have rewritten all the table names formally. We have added a hypothesis test note under each table.

For example: Table 5. The hypothesis being tested is that H1: MP_t-2_ x Debt to assets increased is significant and negative

- Write the limitations of the study and suggestions for future studies.

We have added this section to the conclusion:

Some limitations to this study require further research to keep building a solid framework on using SAFE data for policy making. This study employs robust standard errors but does not investigate the rationale for this standard error treatment. Other standard error treatments, such as clustering at the country level, may be more appropriate as these regressions combine the aggregate effect of monetary policy on micro units by merging aggregate data with micro-observations from SAFE (93). Further, this study does not employ firm fixed effects - in line with the majority of literature which uses SAFE - given the cross-sectional nature of the SAFE firm data. Using firm fixed effects would further isolate the impact of UMP on SME bank liquidity limitations by absorbing any firm-specific credit demand shocks. The appropriate use of standard errors and firm fixed effects is worthy of further investigation.

- There are frequent references to Finnegan and Kapoor in the study. Is this an extension study? If so, state this in the study, if not, state this clearly in the literature section and state that you have followed this study in other literature. This is a blind review, so it is seen as an extension since the identity of the authors is known and this study is also the authors.

Yes, it is an extension study of Finnegan and Kapoor (2023) and this is now stated clearly in the introduction and methodology sections. The research study builds on the mentioned research paper as it provides a good framework for data analysis and testing of outlined hypotheses.

Introduction: It is an extension study on the recent work by Finnegan and Kapoor (1), which compares their probit model to an LPM and logit model.
Methodology: . In particular, it in an extension of the study by Finnegan and Kapoor (1) - which studies the impact of unconventional monetary policy on SMEs' access to bank finance - in terms of assessing the methodological framework when using binary dependent variables and SAFE

Reviewer #2: In my opinion, the paper offers a good contribution. So, I recommend accepting this paper, but after making the following modifications:

Dear Reviewer #2, thank you very much for your valuable comments which we have taken on board to strengthen the paper. We address each one in detail below.

1- I think that the abstract needs improvement.

We have expanded the abstract to describe the main objective of the study and summarise the most important results and their significance. The revised abstract is as follows:

Abstract
Research on small and medium-sized enterprises (SMEs) access to bank finance is vital for the euro area economy. SMEs heavily represent the European business sector, employing around 100 million people and accounting for more than half of Gross Domestic Product. Research studies in the field often rely on the ECB/EC Survey on the Access to Finance of Enterprises (SAFE). Many studies employ probit or logit models with categorical dependent variables derived from SAFE. The research findings show that hardly any study employs the simpler linear probability model (LPM), with a dominant lack of research providing evidence that justifies the model selection process and suitability. However, it is well known that different econometrics models can lack consistency and frequently yield different results. Yet, the literature has no consensus on the best econometric approach. In addition, there is a lack of robustness tests in the literature to ensure model validity, underlining the need for a comprehensive review of the methodological framework that dominates SAFE data use. This paper addresses the identified research gap by introducing a robust methodological framework that helps researchers identify and choose an appropriate categorical model when using SAFE data. The study adds significant value to the extant literature by identifying four criteria that need to be considered when selecting the appropriate model among three common binary dependent models: LPM, probit and logit models.

The findings show that the probit model was appropriate is all cases but that the LPM should not be disregarded, as it can be used in two cases: when considering the interaction between monetary policy and debt to assets and monetary policy and innovation. The use of the LPM is justified as a less complex econometric model, allowing for clearer communication of the results. This innovative, robust approach to choosing the appropriate econometric categorical dependent model when employing SAFE data contributes to support policy effectively.

2- Some equations need correction. Moreover, in some equations, there are some symbols that are not defined.

Hypothesis 1 (H1): UMP decreases the probability of firms with increased debt-to-assets being credit constrained.

To test H1, they model Equation 1 as follows:

$$P\left( {CreditConstrained}_{i,c,t}=1 | x \right)=\alpha_{i}+\beta^{'}{MP}_{c,t-2}+\gamma^{'}{DebttoAssets}_{i,c,t}*{MP}_{c,t-2}+\delta^{'}X_{i,c,t}+\theta^{'}{Macro}_{c,t-2}+\varphi^{'}{BankCh}_{c,t-2}+\tau_{c,s}+\varepsilon_{i,s,c,t}$$

Hypothesis 2 (H2): UMP reduces the probability of risky firms being credit constrained

To test H2, they model Equation 2 as follows:

$$P\left( {CreditConstrained}_{i,c,t}=1 | x \right)=\alpha_{i}+\beta^{'}{MP}_{c,t-2}+\gamma^{'}{FrimRisk}_{i,c,t}*{MP}_{c,t-2}+\delta^{'}X_{i,c,t}+\theta^{'}{Macro}_{c,t-2}+\varphi^{'}{BankCh}_{c,t-2}+\tau_{c,s}+\varepsilon_{i,s,c,t},$$

We include table S1 to ensure that all symbols in the equation are defined.

| Variable | Data Source | Definition |
| --- | --- | --- |
| **Dependent variable** |  |  |
| $\boldsymbol{P}\left( \boldsymbol{CreditConstrained}_{\boldsymbol{i,c,t}}\boldsymbol{=1} \vert\boldsymbol{x} \right)$ | ECB/EC Survey on the access to finance of enterprises (SAFE)  Q7a, Q7b | Binary variable = 1 if the firm reported (i) to have applied for bank loans in the previous six months but was rejected (Credit Denied) or (ii) to have applied but received less than 75% of its demand (Rationed) or (iii) to have refused credit because it was offered at a too high cost (Refused due to high cost) or (iv) not to have applied because of possible rejection (Discouraged). 0 = if the firm reported (i) to have applied for bank loans in the previous six months and they received everything or (ii) they received 75 percent or more of their demand. |
| **Independent variable H1** |  |  |
| $\mathbf{H}\boldsymbol{1}\boldsymbol{\gamma}^{\boldsymbol{'}}\boldsymbol{DebttoAssets}_{\boldsymbol{i,c,t}}\boldsymbol{*}\boldsymbol{MP}_{\boldsymbol{c,t-2}}$ |  |  |
| Debt to Assets increased | ECB/EC SAFE Q2 | = 1 if the firm’s debt to assets increased in the past 6 months, 0 if it remained unchanged or decreased |
| MPc,t-2 = ECB BS Assets (million) | ECB Statistical Data Warehouse | Continuous variable, monthly, total ECB assets (after subtracting the autonomous factors that are beyond the direct control of the ECB including banknotes in circulation and government balances) from individual central bank balance sheet for stressed countries, following Peydro et al. (2021). Monthly data averaged over half years ending in March and September. |
|  |  |  |
| **Independent variable H2** |  |  |
| **H2** $\boldsymbol{MP}_{\boldsymbol{c}\boldsymbol{,}\boldsymbol{t}\boldsymbol{-}\boldsymbol{2}}\boldsymbol{+}\boldsymbol{\gamma}^{\boldsymbol{'}}\boldsymbol{FirmRisk}_{\boldsymbol{i}\boldsymbol{,}\boldsymbol{c}\boldsymbol{,}\boldsymbol{t}}$ |  |  |
| Where ${FirmRisk}_{i,c,t}$*=* |  |  |
| Profit decreased | ECB/EC SAFE Q2 | = 1 if the firm's profit decreased in the past 6 months, 0 if it remained unchanged or increased |
| Credit history deteriorated | ECB/EC SAFE Q11 | = 1 if the firm's credit history deteriorated in the previous 6 months, 0 if it remained the unchanged or improved |
| Own capital deteriorated | ECB/EC SAFE Q11 | = 1 if the firm's own capital deteriorated in the previous 6 months, 0 if it remained unchanged or improved |
| Own outlook deteriorated | ECB/EC SAFE Q11 | = 1 if the firm's own outlook deteriorated in the previous 6 months, 0 if it remained unchanged or improved |
| Innovation | ECB/EC SAFE Q1 | = 1 if the innovated (in terms of new or improved product, new or improved production process, new organisation of management, new way of selling goods or services) in the previous 6 months, 0 if did not |
| $\boldsymbol{X}_{\boldsymbol{i,c,t}}$ **= Firm controls** |  |  |
| Micro | ECB/EC SAFE  QD1 | = 1 if the firm has between 1 and 9 employees, 0 otherwise |
| Micro | ECB/EC SAFE  QD1 | = 1 if the firm has between 1 and 9 employees, 0 otherwise |
| Small | ECB/EC SAFE  QD1 | = 1 if the firm has between 10 and 49 employees, 0 otherwise |
| Medium | ECB/EC SAFE  QD1 | = 1 if the firm has between 50 and 249 employees, 0 otherwise |
| More than 10 years | ECB/EC SAFE  QD5 | = 1 if the firm is 10+ years old, 0 otherwise |
| Between 5 and 10 yrs | ECB/EC SAFE  QD5 | = 1 if the firm is between 5 and 10 years old, 0 otherwise |
| Between 2 and 5 yrs | ECB/EC SAFE  QD5 | = 1 if the firm is between 2 and 5 years old, 0 otherwise |
| Less than 2 yrs | ECB/EC SAFE  QD5 | = 1 if the firm is less than 2 years old, 0 otherwise |
| Stand-alone firm | ECB/EC SAFE  QD2 | = 1 if the firm is an autonomous profit-oriented enterprise, 0 otherwise |
| Individual or family owned | ECB/EC SAFE  QD2 | = 1 if the firm's owner is an individual or a family, 0 otherwise |
| Turnover up to 2mn | ECB/EC SAFE  QD4 | = 1 if the firm's annual turnover is less than €2 mln, 0 otherwise |
| Turnover between 2 and 10mn | ECB/EC SAFE  QD4 | = 1 if the firm's annual turnover is between €2 mln. and €10 mln, 0 otherwise |
| Turnover between 10 and 50mn | ECB/EC SAFE  QD4 | = 1 if the firm's annual turnover is between €10 mn. and €50 mn, 0 otherwise |
| Industry | ECB/EC SAFE  QD3 | = 1 if the firm's main activity is in industry, 0 otherwise |
| Construction | ECB/EC SAFE  QD3 | = 1 if the firm's main activity is in construction, 0 otherwise |
| Wholesale or Retail Trade | ECB/EC SAFE  QD3 | = 1 if the firm's main activity is in wholesale or retail trade, 0 otherwise |
| Services | ECB/EC SAFE  QD3 | = 1 if the firm's main activity is services, 0 otherwise |
| $\boldsymbol{Macro}_{\boldsymbol{c,t-2}}$***= Macroeconomic controls*** |  |  |
| Unemployment | Eurostat | Continuous variable, unemployment rate (share of active population), seasonally adjusted. The unemployment rate is the number of people unemployed expressed as a share of the labour force. The labour force is the total number of people employed and unemployed. Quarterly data averaged over half years ending in March and September and expressed as decimals. |
| Inflation | Eurostat | Continuous variable, inflation rate measured by HICP monthly data (annual rate of change). Average of monthly inflation rate data over half years ending in March and September and expressed as decimals. |
| $\boldsymbol{BankCh}_{\boldsymbol{c,t-2}}\boldsymbol{=}$***Bank controls*** |  |  |
| Non-performing loans | IMF Financial Soundness Indicators | Continuous variable, quarterly non-performing loans as a share of total gross loans averaged over half years ending in March and September and expressed as decimals. |
| Regulatory capital to risk-weighted assets ratio | IMF Financial Soundness Indicators | Continuous variable, quarterly regulatory tier 1 capital as a share of risk weighted assets averaged over half years ending in March and September and expressed as decimals. |

OR

We also clarify these definitions in the text of section 4.1 empirical model to define each symbol in the definition. See changes below:

This dependent variable ‘credit constrained’ equals 1 if the firm reported to have (i) applied for bank loans in the previous six months but was rejected (Credit Denied) or (ii) applied but received less than 75% of its demand (Rationed) or (iii) refused credit because it was offered at too high a cost (Refused due to high cost) or (iv) not applied because of possible rejection (Discouraged). Alternatively, the variable equals 0 if the firm reported to have applied for bank loans in the previous six months and received everything or received 75% and above. Given that the indicator is equal to 1 if the firm is credit constrained, a negative coefficient indicates that SMEs are less likely to be credit constrained.

MP_t−2_ is the one-year lag (equivalent to two survey waves in SAFE) of the logarithm the assets of individual central bank balance sheets - minus autonomous factors - for stressed countries.

H1. Debt-to-assets increased is a categorical variable which equals 1 if the firm’s debt-to-assets increased, and 0 if it remained the same or decreased in the previous six months.

H2. ${FirmRisk}_{i,c,t}$is modelled categorically and the measures for firm risk include a future predictor of risk - profit decreased in the previous six months, as well as a selection of subjective measures of risk - firms own view if there has been deterioration in credit history, own economic outlook and own capital in the previous six months and finally, an activity-based measure of risk - innovative activity given that such activity is more uncertain and therefore riskier.

3- Authors should test multicollinearity and outliers in the data before estimating the model. This point is very important for this study. Furthermore, adding references addressed the two problems in the LPM, logit, and probit models.

Thank you very much for this comment. We agree that this is very important for validity of results. We have therefore added a new section 5.3 on ‘multicollinearity and outliers’ to address this concern. We have also updated Tables 5-10 with measures of multicollinearity and results if outliers are excluded. New section below:

## 5.3 Multicollinearity and outliers

In the SAFE literature, there is barely any discussion on checks for multicollinearity and no discussion of outliers. In terms of multicollinearity, Mac an Bhaird (25), Calabrese et al. (12) and Sclip (11) (11.6% of studies) use the Variance Inflation Factor (VIF) while Mol-Gómez-Vázquez et al. (37) use correlation matrices to identify if there is correlation between the independent variables. There is no discussion of outliers, yet, SAFE is a cross-sectional survey conducted across EU countries, each experiencing different economic cycles over time. It is well known that either phenomenon can distort regression results, and this study checks to ensure that neither multicollinearity or outliers influence the results. In this study, correlation tables are used to identify pairwise correlations. The mean Variance Inflation Factor (VIF) is used to identify the extent to which a given explanatory variable can be explained by all the other explanatory variables in the equation.

The correlation tables in Tables S7-S12 indicate that, in general, pairwise correlation is not present to any great degree with no correlation in excess of 0.8, which is considered acceptable (19, 84, 85). However, the correlation tables show perfect multicollinearity between the interaction term and its constituent parts. This is because there is structural multicollinearity in the model as the key variable of interest for H1 and H2 is an interaction term: H1: $\gamma^{'}{DebttoAssets}_{i,c,t}*{MP}_{c,t-2}$and H2: $\gamma^{'}{FirmRisk}_{i,c,t}*{MP}_{c,t-2}$. The Variance Inflator Factor (VIF) also reflects this structural multicollinearity. Tables 5-10 shows that when the interaction term is present the VIF is between 22 and 28 (Tables 5-10). However, when the interaction term is excluded, the VIF falls below 5, which is considered to indicate an acceptable level of multicollinearity (53, 86, 87).

One solution to interaction terms and structural multicollinearity cited in the literature is to mean centre a constituent variable (standardise the variable by subtracting the mean) (27, 53, 87). However, this is not possible with a binary variable like $DebttoAssets$ or $FirmRisk$. Another solution is to increase the sample size as logit and probit regression uses maximum likelihood estimation (MLE), which relies on large-sample asymptotic normality. This means that the reliability of estimates increases when the sample size is large enough (17, 18, 88). However, the sample size (11,319, which drops to 8,777 on account of the monetary policy lag employed) cannot be increased as it is determined by the research focus on stressed countries and credit-constrained SMEs from 2014-2019.

This study acknowledges and underlines the necessary degree of multicollinearity arising from the interaction term due to its importance in addressing the hypothesis. This is justified because standard errors are relatively small, and the estimates do not change much when more variables are added to the model (Tables S2 -S6), indicating that multicollinearity does not compromise the model (19, 61, 89)

In terms of outliers, there is no discussion in the SAFE literature, despite SAFE being a cross-sectional survey across EU countries with different countries experiencing different economic cycles over time. A single observation that is substantially different from all other observations can make a significant difference in the regression analysis results (19, 53, 90). Studies using SAFE need to consider outliers explicitly, and this paper identifies outliers for each regression and runs the regressions, excluding the outliers to identify if the estimates change. In particular, this study takes the following three steps to identify if the outliers distort the estimates:

1. Identifies the number of outliers - observations with deviance residuals > |3| and their average (91, 92).^[1]^

2. Plots the deviance residuals against the predicted values for credit constrained to identify any extreme values

3. Run the regressions without the observations with deviance residual > |3|

In terms of outliers, Tables 5-10 show the estimates for the interaction terms for both H1 and H2 when observations with a deviance residual > |3|are excluded; the main results are very similar, which ensures confidence in the results (91). The next section concludes the research study.

4- The authors should put the dataset used of this study in the “Supporting Information File".

The authors signed a confidential agreement with the European Central Bank to protect the confidentiality of the SAFE data. However, the data underlying the results presented in the study are available from the European Central Bank in an anonymised format. <https://www.ecb.europa.eu/stats/ecb_surveys/safe/html/index.en.html>. Other data is publicly available from the ECB Statistical Data Warehouse, Eurostat and the International Monetary Fund. This data is uploaded under supporting information file S13.

A data availability statement is completed in the submission form.

5- At the conclusion of this work, limitations of this research should be mentioned.

Thank you for this comment. We have added this section to the conclusion:

Some limitations to this study require further research to keep building a solid framework on using SAFE data for policy making. This study employs robust standard errors but does not investigate the rationale for this standard error treatment. Other standard error treatments, such as clustering at the country level, may be more appropriate as these regressions combine the aggregate effect of monetary policy on micro units by merging aggregate data with micro-observations from SAFE (93). Further, this study does not employ firm fixed effects - in line with the majority of literature which uses SAFE - given the cross-sectional nature of the SAFE firm data. Using firm fixed effects would further isolate the impact of UMP on SME bank liquidity limitations by absorbing any firm-specific credit demand shocks. The appropriate use of standard errors and firm fixed effects is worthy of further investigation.

6. PLOS authors have the option to publish the peer review history of their article ([what does this mean?](https://eur05.safelinks.protection.outlook.com/?url=https%3A%2F%2Fjournals.plos.org%2Fplosone%2Fs%2Feditorial-and-peer-review-process%23loc-peer-review-history&data=05%7C02%7Cd20128420%40mytudublin.ie%7Cb12d624c480149828df908dc5d0f1f7e%7C766317cbe9484e5f8cecdabc8e2fd5da%7C0%7C0%7C638487568248819288%7CUnknown%7CTWFpbGZsb3d8eyJWIjoiMC4wLjAwMDAiLCJQIjoiV2luMzIiLCJBTiI6Ik1haWwiLCJXVCI6Mn0%3D%7C0%7C%7C%7C&sdata=f%2Bx5FIDDutUuzQm5gjvJS%2BauJJa%2FWEE1itoCSLLg6b4%3D&reserved=0)). If published, this will include your full peer review and any attached files.

If you choose “no”, your identity will remain anonymous but your review may still be made public.

We choose ‘yes’ to make the peer reviews available as this adds to the transparency of the research and the comments are valuable to improving the research paper.
General

1. Please ensure that your manuscript meets PLOS ONE's style requirements, including those for file naming. The PLOS ONE style templates can be found at 
https://journals.plos.org/plosone/s/file?id=wjVg/PLOSOne_formatting_sample_main_body.pdf and 
<https://journals.plos.org/plosone/s/file?id=ba62/PLOSOne_formatting_sample_title_authors_affiliations.pdf>

This is done

2. Please ensure that you include a title page within your main document. We do appreciate that you have a title page document uploaded as a separate file, however, as per our author guidelines (http://journals.plos.org/plosone/s/submission-guidelines#loc-title-page) we do require this to be part of the manuscript file itself and not uploaded separately.
Could you therefore please include the title page into the beginning of your manuscript file itself, listing all authors and affiliations.

The amended title page is now included in the main document.

3. Please provide a complete Data Availability Statement in the submission form, ensuring you include all necessary access information or a reason for why you are unable to make your data freely accessible. If your research concerns only data provided within your submission, please write "All data are in the manuscript and/or supporting information files" as your Data Availability Statement.

The authors signed a confidential agreement with the European Central Bank to protect the confidentiality of the SAFE data. However, the data underlying the results presented in the study are available from the European Central Bank in an anonymized format. <https://www.ecb.europa.eu/stats/ecb_surveys/safe/html/index.en.html>. Other data is publicly available from the ECB Statistical Data Warehouse, Eurostat and the International Monetary Fund. This data is uploaded under supporting information file S13.

A data availability statement is completed in the submission form.

4. Please include captions for your Supporting Information files at the end of your manuscript, and update any in-text citations to match accordingly. Please see our Supporting Information guidelines for more information: http://journals.plos.org/plosone/s/supporting-information.

Done

1.	Caglio C, Darst M, Kalemli-Ozcan S. *Risk-Taking and Monetary Policy Transmission: Evidence from Loans to SMEs and Large Firms.* CEPR Discussion Paper, No. DP17174. 2022.

2.	Durante E, Ferrando A, Vermeulen P. *Monetary policy, investment and firm heterogeneity*, European Central Bank: Working Paper Series, No.2390. 2020.

3.	Ferrando A, Popov A, Udell GF. Unconventional monetary policy, funding expectations, and firm decisions. European Economic Review. 2022 2022/10/01/;149:104268.

4.	Albertazzi U, Becker B, Boucinha M. Portfolio rebalancing and the transmission of large-scale asset purchase programs: Evidence from the Euro area. Journal of Financial Intermediation. 2021 2021/10/01/;48:100896.

5.	Moulton BR. An Illustration of a Pitfall in Estimating the Effects of Aggregate Variables on Micro Units. The Review of Economics and Statistics. 1990;72(2):334-8.

6.	Finnegan M, Kapoor S. ECB unconventional monetary policy and SME access to finance. Small Business Economics. 2023 2023/02/18.

1. This is because deviance residuals can be roughly approximated with a standard normal distribution when the model holds (Agresti, 2013). Residuals greater than the absolute value of 3 are in the tails of a standard normal distribution and this usually indicates strain in the model (Ford, 2022).
